# Programmed Design of a Lithium–Sulfur Battery Cathode by Integrating Functional Units

**DOI:** 10.1002/advs.201900711

**Published:** 2019-07-19

**Authors:** Zhipeng Zeng, Wei Li, Qiang Wang, Xingbo Liu

**Affiliations:** ^1^ Department of Mechanical and Aerospace Engineering West Virginia University Morgantown WV 26506 USA; ^2^ Department of Physics and Astronomy West Virginia University Morgantown WV 26506 USA; ^3^ Shared Research Facilities West Virginia University Morgantown WV 26506 USA

**Keywords:** chemical anchors, electrocatalysis, integrated electrodes, lithium–sulfur batteries, polysulfides

## Abstract

Sulfur is considered to be one of the most promising cathode materials due to its high theoretical specific capacity and low cost. However, the insulating nature of sulfur and notorious “shuttle effect” of lithium polysulfides (LiPSs) lead to severe loss of active sulfur, poor redox kinetics, and rapid capacity fade. Herein, a hierarchical electrode design is proposed to address these issues synchronously, which integrates multiple building blocks with specialized functions into an ensemble to construct a self‐supported versatile cathode for lithium–sulfur batteries. Nickel foam acts as a robust conductive scaffold. The heteroatom‐doped host carbon with desired lithiophilicity and electronic conductivity serving as a reservoir for loading sulfur can trap LiPSs and promote electron transfer to interfacial adsorbed LiPSs and Ni_3_S_2_ sites. The sulfurized carbon nanofiber forest can facilitate the Li‐ion and electron transport and retard the LiPSs diffusion as a barrier layer. Sulfiphilic Ni_3_S_2_ acts as both a chemical anchor with strong adsorption affinity to LiPSs and an efficient electrocatalyst for accelerating kinetics for redox conversion reactions. Synergistically, all functional units promote the lithium ion coupled electron transfer for binding and redox conversion of LiPSs, resulting in high reversible capacities, remarkable cycle stability, and excellent rate capability.

## 1 Introduction

To satisfy the ever‐increasing demands for the portable electronic devices, electric vehicles, and renewable energy harvesting at a large scale, intensive research efforts have been devoted to exploring reliable energy storage systems with high energy densities and low cost.^1^ The lithium–sulfur (Li–S) battery, based on conversion reaction chemistry between Li anode and S cathode, is considered as one of the promising candidates due to its high theoretical energy density (≈2600 W h kg^−1^). Sulfur can provide a high theoretical capacity (≈1600 mAh g^−1^) and has various merits of high natural abundance, low cost, environmental benignity, and nontoxicity.^2^ However, the practical application for Li–S batteries is still hindered by a series of technical issues, including the insulating nature of sulfur (5 × 10^−30^ S cm^−1^ at 25 °C) as well as the discharged products (Li_2_S/Li_2_S_2_), large volume change (≈80%) during the charge/discharge process, and the most critical problem of dissolution of lithium polysulfides (LiPSs) into electrolytes and their “shuttle effect.”^3^ During the lithiation of S cathode, the long‐chain high‐polarity LiPSs intermediates (Li_2_S*_x_*, 4 ≤ *x* ≤ 8) are formed and easily dissolved in the liquid electrolyte and tend to migrate to the Li anode side, causing parasitic reactions. Particularly, the low conductivity of S and Li_2_S/Li_2_S_2_ and the high solubility and diffusion of LiPSs lead to high charge transfer resistance and sluggish kinetics of polysulfide redox reactions on the cathode. Collectively, these issues likely result in low utilization of sulfur, loss of active materials, low coulombic efficiency and redox kinetics, structural collapse, and degradation of electrodes and thus impair the capacity, rate capability, and cycling stability of Li–S batteries.^4^

Confining sulfur within various host materials has been a common strategy to reduce the diffusion of LiPSs for the cathode design. Early efforts have focused on developing conductive hollow and porous carbon hosts to physically immobilize the LiPSs, alleviate the volume changes and enhance the conductivity, bringing progressive enhancement of composite sulfur/carbon cathode performance.^5^ However, the high polarity of polysulfides reduces their affinity toward nonpolar carbon hosts. Their physical interaction is primarily based on relatively weak van der Waals' force, which cannot entirely prevent the transport of LiPSs in the long term especially for high loading of sulfur, since the driving force for the migration of LiPSs is a much stronger electric field in Li–S batteries.^6^ Furthermore, the incompatibility in the surface affinity also impedes the efficient interfacial redox reaction of sulfur species on carbon, possibly causing flooding of polysulfides. Therefore, recent attention has been paid to seek polar host materials with adequate strong chemical binding affinity to polysulfides. Diverse materials such as heteroatom‐doped carbon,[qv: 2a,7] polymer chains,^8^ and various transition‐metal compounds including metal oxides,[qv: 6b,9] sulfides,[qv: 3c,10] nitrides,^11^ and carbides,^12^ have been developed with varied affinity to polysulfides and conductivity. Among them, metal sulfides have attracted particular interest due to their strong sulfiphilicity, tunable crystal structures, and stoichiometric compositions.^10^, ^13^ In addition, some of the metal sulfides have been demonstrated to have an electrocatalytic effect in improving polysulfide redox reactions. For instance, Co_9_S_8_,^14^ MoS_2_,[qv: 13c,15] NiS_2_,^16^ and TiS_2_ 17 have been used as sulfur hosts for Li–S batteries with enhanced binding affinity to LiPSs. However, most of the metal sulfides have low electronic conductivity compared to carbonaceous materials and easily aggregate to form large particles, thus limiting the charge transfer, surface area, adsorption site density, and high loading of sulfur species. For example, Li et al. introduced a moderate amount of elemental sulfur loaded on the surface of dense Ni_3_S_2_ layers on the Ni foam, forming a 3D hierarchical Ni/Ni_3_S_2_/S electrode. However, the electrode showed limited cycle life with low coulombic efficiency due to the poor adsorption mechanism toward polysulfides.^18^ To enhance the conductivity and loading of sulfur, carbonaceous materials have been combined with metal sulfides either in a mixture powder or freestanding aerogel manner.[qv: 13c,15a,16] The former relies on the utilization of polymers binders and limits the charge transfer between particles, while the latter suffers from the mechanically fragile scaffold structure of aerosol and may collapse after long‐term cycles. Therefore, it is still challenging to develop binder‐free monolithic cathodes with high conductivity, favorable interfacial interaction, strong capability for trapping LiPSs, a large density of adsorption sites, and fast redox kinetics for high‐energy Li–S batteries.

**Figure 1:**
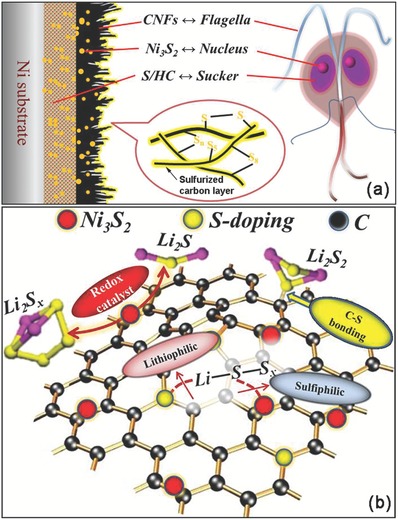
Schematic illustration of a) the flagellate‐like S/CNF‐HC‐Ni_3_S_2_ cathode and giardia lamblia. b) The mechanism of cooperative lithiophilic and sulfiphilic interfaces of S‐doped carbon and Ni_3_S_2_ for enhanced adsorption and electrocatalytic conversion of lithium polysulfides.

Herein, a bioinspired electrode structure design is proposed to construct self‐supported cathodes (S/CNF‐HC‐Ni_3_S_2_) integrating Ni foam framework, host carbon (HC), carbon nanofibers (CNFs), and Ni_3_S_2_ with active sulfur for high‐performance Li–S batteries through a programmed fabrication approach. As illustrated in **Figure** **1**a, the architecture of such cathode mimics the structure of giardia lamblia, a parasitic microorganism. Two kinds of carbon layers are in situ grown on Ni foam framework, consisting of S‐doped host carbon and carbon nanofiber forest, corresponding to the “sucker” and “flagella,” respectively. Considerable Ni_3_S_2_ nanoparticles are uniformly distributed in the carbon matrix, similar to the “nucleus.” The HC layer serves as the primary reservoir for loading of sulfur. As the giardia lamblia shows strong adhesion ability to the surface of the infected hosts, this electrode with similar structure design is expected to have affinity toward LiPSs species. In this work, such elaborately designed cathode possesses cooperative interfaces of “lithiophilic” S‐doped carbon and “sulfiphilic” Ni_3_S_2_ (Figure 1b). The Ni_3_S_2_ particles have strong chemical adsorption affinity to polysulfide and high electrocatalytic activity for facilitating the LiPSs‐involved redox reactions. The HC layer can enable a relatively high loading of sulfur and the partially sulfurized CNF layer can act as a barrier/functional layer to prevent the diffusion of LiPSs and facilitate the transport of Li ions and electrons, both of which can also accommodate the volume changes. Overall, this hierarchical electrode design integrates multiple building blocks with specialized roles into an ensemble to show a synergistic effect, providing a firm and effective 3D conductive network and cooperative interfaces to minimize the shuttle effect by increasing the density of adsorption sites, adsorption capability, electron/ion transfer, and catalytic redox kinetics for the sulfur species during the discharge–charge process. As a result, the S/CNF‐HC‐Ni_3_S_2_ cathode exhibits a stable reversible capacity of ≈850 mAh g^−1^ after 100 cycles at a current density of 0.2 C, excellent rate capability, and superior cycle durability (620 mAh g^−1^ after 300 cycles at 2 C and 400 mAh g^−1^ after 500 cycles at 5 C). This work offers a programmed design strategy by integrating hierarchical functional units to develop high‐performance cathodes for Li–S batteries.

## 2 Results and Discussion

**Figure 2:**
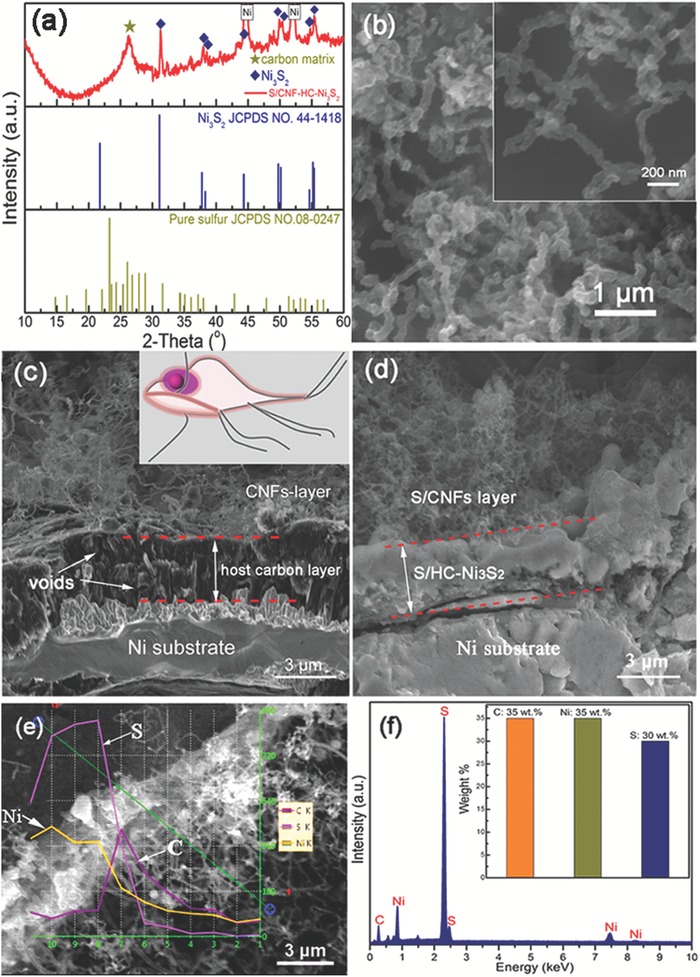
a) The magnified XRD pattern of S/CNF‐HC‐Ni_3_S_2_ and standard XRD cards of Ni_3_S_2_ and S. SEM images of the top view of b) S/CNF‐HC‐Ni_3_S_2_ electrode. Cross‐sectional SEM images of c) Ni foam after the CVD process and d) S/CNF‐HC‐Ni_3_S_2_ electrode. Inset of (c) is a lateral view of giardia lamblia structure. e) EDX line scan profile and f) EDX spectrum of S/CNF‐HC‐Ni_3_S_2_ with the corresponding elemental quantification in the inset.

The programmed fabrication process of S/CNF‐HC‐Ni_3_S_2_ electrodes includes the hydrothermal surface pretreatment of Ni foam (Figures S1 and S2, Supporting Information), chemical vapor deposition (CVD) for growth of host carbon and carbon nanofiber forest and stepwise thermal treatment for Ni_3_S_2_ and sulfur incorporation (Figures S3 and S4, Supporting Information) followed by CS_2_ rinsing to remove bulk S residues. The average S mass ratio is estimated to 8 wt% by thermal gravimetric analysis (TGA, Figure S5, Supporting Information), which coincides with the mass change results as listed in Table S1 of the Supporting Information. The dominant peaks in the X‐ray diffraction (XRD) pattern of cleaned S/CNF‐HC‐Ni_3_S_2_ correspond to metallic nickel resulting from Ni foam framework (Figure S6a, Supporting Information), and the XRD patterns of other components are compared in Figure S6b of the Supporting Information. Further magnification of the XRD pattern of cleaned S/CNF‐HC‐Ni_3_S_2_ (**Figure** **2**a) can unambiguously identify the existence of many minor peaks of the rhombohedral Ni_3_S_2_ phase (JCPDS No. 44–1418) and a broad peak centered at 26.4° arising from the graphitic carbon matrices with sulfur. One sharp peak at 2θ ≈ 22° for Ni_3_S_2_ somehow disappears, which is probably due to the broad hump and ascending background from the graphitic carbon overlapping with it. Few sharp peaks of crystalline sulfur can be observed, indicating that sulfur was well dispersed in the carbon layer of the cathode. The morphologies of Ni foam substrate and S/CNF‐HC‐Ni_3_S_2_ cathode were characterized by the scanning electron microscopy (SEM) at different stages of preparation process. After the CVD process, the smooth surface of Ni foam is coated with numerous carbon nanofibers (Figure S7, Supporting Information). Then after stepwise thermal treatment for Ni_3_S_2_ and sulfur incorporation, the morphology of CNFs has little change except for the slight coarsening (Figure 2b). Few agglomerations of bulk sulfur particles can be observed suggesting the homogeneous dispersion and loading of sulfur within the S/CNF‐HC‐Ni_3_S_2_, consistent with the XRD results. The energy dispersive X‐ray (EDX) elemental mapping of the ligament surface of S/CNF‐HC‐Ni_3_S_2_ demonstrates that Ni and S elements are homogeneously dispersed in the carbon matrix without notable segregation (Figure S8, Supporting Information). To clearly reveal the hierarchical architecture of Ni foam subjected to CVD treatment and cleaned S/CNF‐HC‐Ni_3_S_2_, their cross‐sectional SEM images are shown in Figure 2c,d, respectively. Three layers can be observed distinctly after CVD process, including the top layer of CNF forest, interlayer of HC with void space, and Ni substrate layer for the Ni foam. S/CNF‐HC‐Ni_3_S_2_ has a similar hierarchical structure, while the HC interlayer is filled with sulfur and in situ formed Ni_3_S_2_ nanoparticles are embedded in the whole matrix. The cross‐sectional EDX line scan profile and spectrum (Figure 2e,f) of S/CNF‐HC‐Ni_3_S_2_ corroborate that sulfur is primarily loaded in the HC reservoir and Ni_3_S_2_ is dispersed in CNF and HC matrix. This is the embodiment of the design that we conceived in Figure 1a.

**Figure 3:**
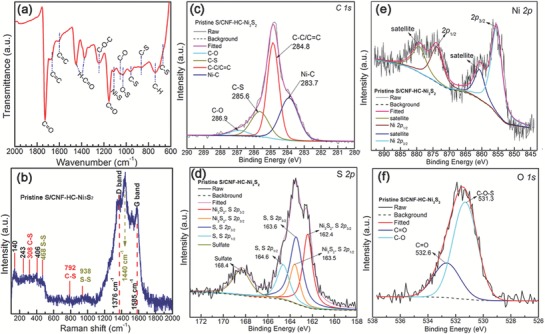
a) FTIR and b) Raman spectra, and high‐resolution XPS spectra of c) C 1s, d) S 2p, e) Ni 2p, and f) O 1s peaks of pristine S/CNF‐HC‐Ni_3_S_2_ electrode.

The chemical interactions within S/CNF‐HC‐Ni_3_S_2_ especially associated with the chemical adsorption toward LiPSs were further investigated by Fourier transform infrared (FTIR), Raman spectroscopy, and X‐ray photoelectron spectroscopy (XPS). In the FTIR spectrum (**Figure** **3**a), the typical peaks at 1575 and 1665 cm^−1^ can be ascribed to the C=C stretching vibrations originating from the graphite planar.^19^ The peak at 1728 cm^−1^ is assigned to symmetric stretching vibrations of —COOH groups on the pyrolysis carbon.[qv: 19b,20] The characteristic peaks at 1330 and 1450 cm^−1^ can be due to the H—C stretching modes of H—C=O in carboxyl groups.^20^ Other oxygen‐containing and CH group can be also identified at the peaks of 1240 (C—O—C), 1052 (C—OH), and 740 cm^−1^ (C—H), respectively.^21^ These oxygen‐containing functional groups can act as active sites bonding with the short sulfur chains, resulting in a peak at 1028 cm^−1^ corresponding to the O—S vibration.^22^ The characteristic peaks of C—S bonds are located at 670 and 955 cm^−1^,^23^ while the peak of Ni—S bond is at 1100 cm^−1^.^24^ In the Raman spectrum (Figure 3b), two prominent peaks at 1376 and 1585 cm^−1^ corresponding to the D (disordered carbon) and G (graphitic carbon) bands are well‐documented in the previous literature.^25^ A sharp peak at ≈1440 cm^−1^ is likely assigned to C—H [δ(CH_2_)] deformation caused by the methyl group from the residual toluene.^26^ Additionally, two small humps centered at the peaks of 792 and 938 cm^−1^ correspond to C—S and S—S vibrations, respectively.^27^ A hump region from 100 to 500 cm^−1^ indicates complicated vibration modes, where the peaks at 140, 243, and 406 cm^−1^, are assigned to the vibrational modes of nickel sulfides.^28^ In addition, the characteristic peaks of C—S and S—S can be identified at 308 and 468 cm^−1^, respectively, indicating that sufficient active anchor sites for chemical adsorption toward polysulfides have been successfully created in this integrated electrode during the sulfurization process,[qv: 8a,27b] as also confirmed by the Raman spectra of different components in Figure S9 of the Supporting Information.

The survey XPS spectrum of pristine S/CNF‐HC‐Ni_3_S_2_ displays the typical peaks for C, S, Ni, and O elements (Figure S10a, Supporting Information). The C 1s XPS spectrum can be deconvoluted into four peaks (Figure 3c). The typical peak at 284.8 and 286.9 eV can be ascribed to sp^2^ hybridized carbon and C—O species, respectively. The peak at 285.6 eV corresponding to C—S bonds proves the covalent bonding between sulfur and carbon matrices in the composite.^29^ A shoulder peak at 283.7 eV corresponds to Ni—C bond resulting from the Ni_3_C formed during the CVD process.[qv: 25b,30] The possible carbon deposition process and stages can be found in Figures S3 and S4 of the Supporting Information. The S 2p spectrum (Figure 3d) demonstrates a broad peak centered at 168.4 eV, which is assigned to the sulfate resulting from the adventitious oxidation of the surface during the sample transfer.[qv: 7c,25e,31] As sulfur and Ni_3_S_2_ are both included in the electrode, there should be two 2p_3/2_/2p_1/2_ doublets in S 2p spectrum. The peaks at 164.6 and 163.6 eV can be attributed to the spin–orbit coupling, but the binding energy of the S 2p_3/2_ peak (163.6 eV) is lower than that of elemental sulfur (164.0 eV), reconfirming the chemical bonding sulfur atoms with carbon matrix (C—S).[qv: 29a,32] The other two peaks at 163.5 eV and 162.4 eV should be reasonably attributed to Ni_3_S_2_, which also coincides with the reported literature.^33^ The Ni 2p_3/2_ and 2p_1/2_ peaks at 856.1 and 873.7 eV are coupled with their satellite peaks at 861.7 and 879.7 eV (Figure 3e), respectively, consistent with those of reported Ni_3_S_2_.^18^, ^34^ This confirms the existence of Ni_3_S_2_ in S/CNF‐HC‐Ni_3_S_2_ along with the XRD results. Figure 3f shows the XPS peak of the O 1s core level of pristine S/CNF‐HC‐Ni_3_S_2_, which is deconvoluted into two peaks. The peak at 532.6 eV can be due to the C=O groups in the aromatic ring; while the other peak at 531.3 eV is due to the C—O bonds. This C—O binding energy is slightly lower than the reported values, indicating that the O atoms are possibly sulfurized to form the C—O—S bonds as functional groups.[qv: 3b,35] Collectively, these morphological, structural and spectroscopic characterization results substantiate that self‐supported S/CNF‐HC‐Ni_3_S_2_ electrode was obtained on the basis of our elaborate design with hierarchical architecture integrating Ni foam framework, carbon host reservoir, carbon nanofiber forest, and Ni_3_S_2_ with active sulfur. Although the functional groups demonstrate the interaction of CNF‐HC‐Ni_3_S_2_ host with sulfur rather than polysulfides, however, it is believed that the formed LiPSs will also chemically interact with the host upon discharge of the bonded sulfur. [qv: 12b]

**Figure 4:**
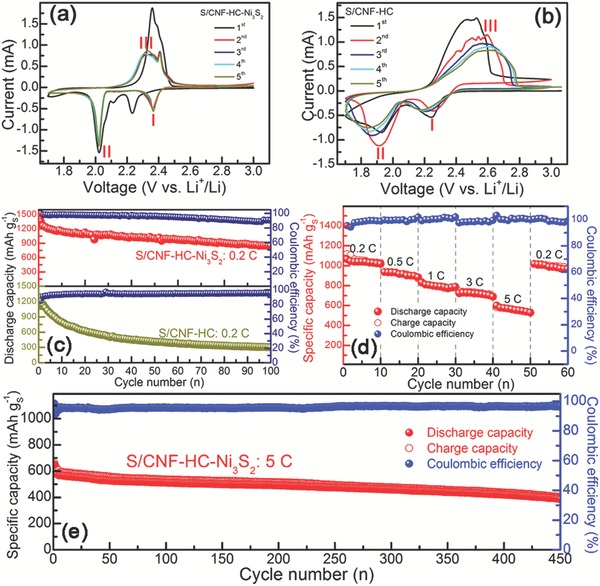
CV curves of a) S/CNF‐HC‐Ni_3_S_2_ and b) S/CNF‐HC cathodes for 5 cycles, c) the cycle performance of S/CNF‐HC‐Ni_3_S_2_ and S/CNF‐HC cathodes at 0.2 C, d) the rate capability, and e) long‐term cycles of S/CNF‐HC‐Ni_3_S_2_ cathode at current density of 5 C for Li–S batteries.

The electrochemical performances of S/CNF‐HC‐Ni_3_S_2_ were systemically investigated as cathodes of Li–S batteries. To study the role of Ni_3_S_2_, S/CNF‐HC (Figure S11, Supporting Information) was also prepared as a control sample with the similar morphology and architecture except for the absence of Ni_3_S_2_ (see details in the Experimental Section). **Figure** **4**a shows the first five cyclic voltammogram (CV) curves of the S/CNF‐HC‐Ni_3_S_2_ electrode at a scan rate of 0.1 mV s^−1^ between 1.7 and 3.0 V. Two sharp cathodic peaks at ≈2.3 (I) and ≈2.0 (II) V can be observed, corresponding to the reduction of S_8_ to long chain LiPSs (Li_2_S*_x_*, 4 ≤ *x* ≤ 8) and then to insoluble short chain discharged products Li_2_S_2_/Li_2_S. Two anodic peaks appear at ≈2.3 and ≈2.4 V, accounting for the oxidation of lithium sulfides to LiPSs and sulfur.^36^ After the initial activation cycle, the following successive CV curves are well overlapped, indicating the highly reversible redox conversion reactions and constant suppression on electrochemical polarization.[qv: 13c,37] By contrast, the S/CNF‐HC electrode exhibits much broader CV peaks for both cathodic and anodic reactions, apparent peak shifts and degradation during continuous cycles (Figure 4b), indicative of slow redox kinetics and severe polarization due to the absence of Ni_3_S_2_ component. For better comparison, the first CV curves of S/CNF‐HC‐Ni_3_S_2_ and S/CNF‐HC are shown in Figure S12 of the Supporting Information. It is obvious that the cathodic and anodic peaks of S/CNF‐HC‐Ni_3_S_2_ electrode are sharper and narrower, which also confirms the critical role of Ni_3_S_2_ during the redox reactions. The CV results signify that Ni_3_S_2_ is able to substantially accelerate kinetics, promote redox reversibility and stability, and mitigate polarization in LiPSs redox reactions.

The cycling performances of S/CNF‐HC‐Ni_3_S_2_ and S/CNF‐HC cathodes at 0.2 C are compared in Figure 4c. The specific capacity of S/CNF‐HC cathode is rapidly decreased to only ≈400 mAh g^−1^ after 100 cycles, whereas the S/CNF‐HC‐Ni_3_S_2_ electrode still maintains a high specific capacity (≈850 mAh g^−1^). This result manifests that the design of S/CNF‐HC‐Ni_3_S_2_ can effectively mitigate the diffusion of soluble LiPSs and loss of active sulfur, minimize the volume change and structural collapse, thus resulting in the high sulfur utilization, specific capacity and cyclability. The corresponding discharge and charge curves (Figure S13, Supporting Information) show characteristic plateaus in good agreement with their respective CV curves as well as the results in the literature.^38^ In sharp contrast to S/CNF‐HC with exacerbated polarization, the S/CNF‐HC‐Ni_3_S_2_ cathode exhibits overlapped discharge/charge voltage stages and low polarization during cycles. This suggests the effective binding and anchoring of LiPSs with abundant active sites, fast redox kinetics, and remarkable reversibility in S/CNF‐HC‐Ni_3_S_2_. The rate capability of S/CNF‐HC‐Ni_3_S_2_ cathode was evaluated at various rates (Figure 4d) with corresponding charge/discharge curves presented in Figure S13c of the Supporting Information. S/CNF‐HC‐Ni_3_S_2_ exhibits stable and high reversible capacities of 1017.8, 883.8, 787.4, and 688.8 mAh g^−1^ at 0.2, 0.5, 1, and 3 C, respectively. Even when current rate increases to 5 C, a capacity of 530 mAh g^−1^ can still be maintained. In addition, the characteristic stable and phased plateaus can still be clearly observed even at 5 C (Figure S13c, Supporting Information), reflecting the enhanced redox kinetics of S/CNF‐HC‐Ni_3_S_2_. Such outstanding rate performance is attributed to the integrated conductive electrode architecture composed of Ni foam, HC and CNF layers for providing 3D electron pathway network and to rich active and adsorption sites for facilitating ion transfer and redox kinetics. The excellent structure stability of S/CNF‐HC‐Ni_3_S_2_ electrode can also be revealed by Figure S14 of the Supporting Information. The stable high capacity could still be maintained for S/CNF‐HC‐Ni_3_S_2_ electrode after the current density switched back from 5 to 0.2 C. A capacity of 810 mAh g^−1^ can be delivered on return to 0.2 C with 80% specific capacity retention after 110 cycles (Figure S14a, Supporting Information), indicating the excellent robustness and stability of the integrated electrode. Moreover, long‐term high rate cycling stability for S/CNF‐HC‐Ni_3_S_2_ electrodes was determined at 2 and 5 C. The capacities can be maintained at ≈620 mAh g^−1^ (2 C) and ≈400 mAh g^−1^ (5 C) after 300 cycles (Figure S14b, Supporting Information) and 450 cycles (Figure 4e), respectively, with the coulombic efficiencies close to 100%. This accentuates the enhanced cycling stability and redox kinetics primarily owing to the structural design advantages. More remarkably, the host carbon layer acting as the main reservoir for sulfur in S/CNF‐HC‐Ni_3_S_2_ can accommodate a relatively high sulfur loading of ≈4 mg cm^−2^, which can sustain a reversible discharge capacity of ≈770 mAh g^−1^ (i.e., 3.2 mAh cm^−2^) at 0.2 C after 100 cycles (Figure S15, Supporting Information), presenting the superior design of S/CNF‐HC‐Ni_3_S_2_ cathodes with great promise in robust, long‐term, and high current load energy applications. Above all, the electrochemical performances of S/CNF‐HC‐Ni_3_S_2_ stand out among recently reported cathodes for Li–S batteries (Table S2, Supporting Information).

**Figure 5:**
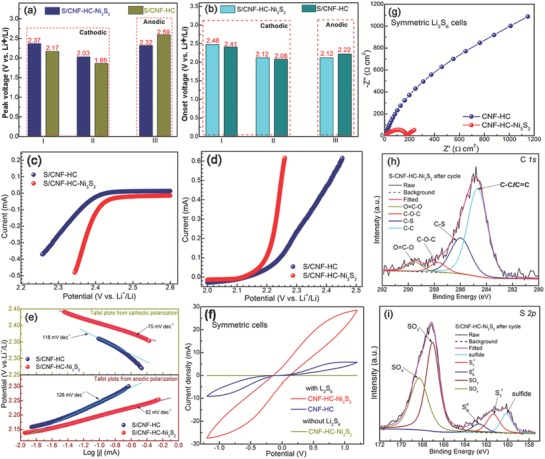
Comparison of a) CV peak voltages, b) onset potentials, c) cathodic and d) anodic polarization curves, and e) Tafel plots of asymmetrical Li–S cells consisting of both S/CNF‐HC‐Ni_3_S_2_ or S/CNF‐HC cathode and Li anode. f) CV curves and g) EIS Nyquist curves of symmetric cells of CNF‐HC‐Ni_3_S_2_ or CNF‐HC electrodes. h) C 1s and i) S 2p XPS spectra of S/CNF‐HC‐Ni_3_S_2_ cathode in asymmetrical Li–S cells after 100 cycles at discharged state.

In‐depth electrochemical experiments and postmortem analyses were performed to anatomize the reasons for the outstanding electrochemical performances of S/CNF‐HC‐Ni_3_S_2_ cathodes, which will shed light on the mechanistic insights for guiding rational and competent cathode designs in the future. First, the well‐distributed Ni_3_S_2_ particles can serve as a highly efficient electrocatalyst with high electrocatalytic activity for both reducing the energy barriers and facilitating the kinetics for LiPSs‐involved redox reactions. To elucidate the electrocatalytic effects, the peak voltages of S/CNF‐HC‐Ni_3_S_2_ and S/CNF‐HC electrodes for two cathodic peaks (I and II) and one anodic peak (III) derived from their CV curves (Figure 4) are compared (**Figure** **5**a). The presence of Ni_3_S_2_ can raise the discharge voltages of cathodic peaks by at least 170 mV and reduce the charge voltage of anodic peak by 270 mV. In this case, Ni_3_S_2_ is able to substantially mitigate the polarization from 0.73 to 0.29 V (i.e., voltage hysteresis between III and II). These results are consistent with those of the galvanostatic discharge–charge profiles (Figure S13, Supporting Information), suggesting that Ni_3_S_2_ can weaken the energy barriers for redox reactions. The onset potential was taken at a current density of 10 µA cm^−2^ beyond the baseline current, determined by a reported method.^14^ Likewise, Ni_3_S_2_ contributes to the increased onset potentials of cathodic peaks (I and II) and decreased onset potential of anodic peak (III) (Figure 5b). Such trends are more evident in the comparison of their polarization curves (Figure 5c,d). Tafel plots were obtained from the polarization curves to uncover the electrocatalytic effect of Ni_3_S_2_ on the charge transfer kinetics in LiPSs‐involved redox reactions. S/CNF‐HC‐Ni_3_S_2_ shows Tafel slopes of 75 and 62 mV dec^−1^ in the reduction (I) and oxidation (III) processes (Figure 5e), respectively, much smaller than those of S/CNF‐HC counterpart, implying the promoted kinetics over the Ni_3_S_2_ electrocatalyst. Moreover, the redox kinetics and charge transfer were examined by CV in symmetric cells, using two identical electrodes with Li_2_S_6_ electrolyte. To eliminate the influence from the capacitive background current in the CV curves, a symmetric cell with the Li_2_S_6_‐free electrolyte and CNF‐HC‐Ni_3_S_2_ electrode couple was also measured, presenting negligible current density (Figure 5f). The current density of CNF‐HC‐Ni_3_S_2_ symmetric cells is much higher than that of CNF‐HC control sample, indicative of notable enhancement on the redox reactions of LiPSs. The Nyquist plots in the electrochemical impedance spectroscopy (EIS) of symmetrical cells further confirm the boosted charge transfer process at Li_2_S_6_/CNF‐HC‐Ni_3_S_2_ interface, with a significantly lower charge transfer resistance (*R*_ct_) of 168 Ω cm^2^ compared to that of CNF‐HC (2214 Ω cm^2^). Detailed equivalent circuit and fitting results are shown in Figure S16 of the Supporting Information. It is clear that the intimate coupling of well‐distributed sulfiphilic Ni_3_S_2_ and electrically conductive HC and CNF matrices can readily promote the access of polysulfide ions and electron transfer to LiPSs/Ni_3_S_2_ interface to trigger the LiPSs redox reactions, unambiguously demonstrating expedited redox conversion kinetics and charge transfer. Collectively, these electrochemical analyses verify that Ni_3_S_2_ plays pivotal roles in electrocatalytically decreasing the energy barriers and accelerate kinetics for LiPSs‐involved redox reactions. Second, the S/CNF‐HC‐Ni_3_S_2_ cathode possesses rich anchoring and adsorption sites with strong chemical LiPSs binding capability for immobilizing soluble LiPSs at the interfacial sites through interactions primarily with polar Ni_3_S_2_ and possible C—S bonds. To unravel the interactions, the XPS characterization of S/CNF‐HC‐Ni_3_S_2_ cathode after 100 cycles at the discharged state was conducted. For C 1s spectrum (Figure 5h), besides the C—C/C=C bonds from the carbon host, a strong peak corresponding to C—S bonding can be identified at 286.0 eV, which is shifted toward higher binding energy by 0.4 eV compared to that of the pristine electrode, together with the emerging carbonate species on the cycled electrode. This signifies the interaction of polysulfide with sulfurized carbon host.^39^ The S 2p spectrum (Figure 5i) shows three major peaks of discharged lithium polysulfide and sulfide on the surface of cycled cathode, centered at 162.7, 161.3, and 160.1 eV, corresponding to the residual bridging ($S_{B}^{0}$), terminal ($S_{T}^{-1}$) sulfur species and sulfides, respectively.^40^ These binding energies are lower than those of pristine counterparts, indicating the chemical trapping of polysulfide/sulfide species on the S/CNF‐HC‐Ni_3_S_2_.[qv: 39b,40c,41] Moreover, the negative shift of the Ni 2p_3/2_ peak together with the Li 1s spectrum is also observed on the cycled electrode (Figure S17, Supporting Information), indicating the chemical bonding between Ni_3_S_2_ and LiPSs.^16^, ^42^

**Figure 6:**
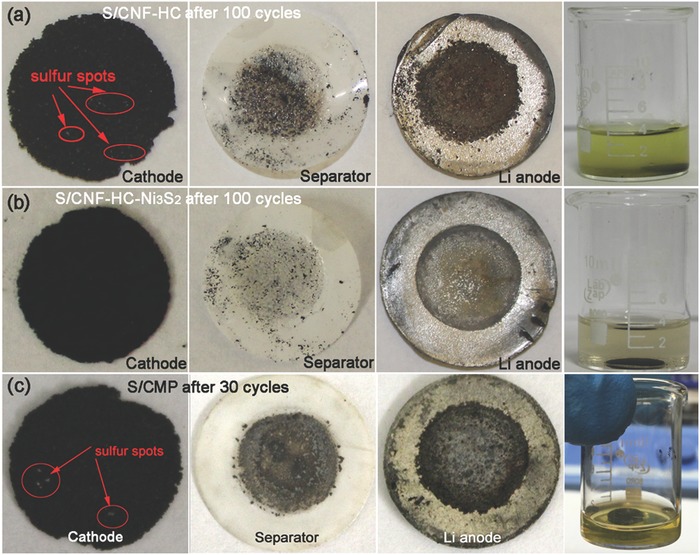
Photographs of the cycled Li–S cells of a) S/CNF‐HC and b) S/CNF‐HC‐Ni_3_S_2_ cathodes after 100 cycles and c) S/CMP after 30 cycles with the corresponding separators, Li anodes, and respective visualized cathodes soaked in dimethoxyethane solvent.

To further visualize the suppressive effects of Ni_3_S_2_ on the shuttling of LiPSs, the pristine electrodes of S/CNF‐HC and S/CNF‐HC‐Ni_3_S_2_ were immerged into 0.1 m Li_2_S_6_ solution for adsorption ability test toward sulfides. Figure S18 of the Supporting Information shows the optical image of the adsorption test result. After standing for 3 days, the solution involved with the S/CNF‐HC‐Ni_3_S_2_ becomes colorless, while it still keeps light yellow for S/CNF‐HC electrode. This comparative result demonstrates the excellent capability of S/CNF‐HC‐Ni_3_S_2_ electrode in polysulfides immobilization. Moreover, **Figure** **6** compares the photographs of cycled Li–S cells based on S/CNF‐HC‐Ni_3_S_2_ and other control samples. The S/CNF‐HC cathode (Figure 6a) contains visible white sulfur spots and yellow green soluble LiPSs solution can be found when the cycled cathode was soaked in dimethoxyethane (DME). Meanwhile, black contaminants adhere to the separator and the Li foil has been seriously corroded, indicating the severe migration of LiPSs toward Li anode due to the weak binding capability of S/CNF‐HC in the absence of Ni_3_S_2_ chemical anchors. Indeed, S/CNF‐HC delivers fast capacity decay during cycles (Figure 4c). Previous reports have also confirmed that the incompatibility in the surface affinity of nonpolar carbon with polar polysulfides makes it incapable of effectively inhibiting LiPSs migration and flooding in pure carbon‐based sulfur cathodes.[qv: 8a,43] In stark contrast, the S/CNF‐HC‐Ni_3_S_2_ electrode (Figure 6b) after 100 cycles shows few visible sulfur species, and its solution is slightly colored when soaked in DME solvent. The separator and Li anode are much cleaner, demonstrating the extraordinary chemical adsorption and binding capabilities for anchoring polysulfides. The binding energies of multiple LiPSs on the Ni_3_S_2_ surface at the molecular level have been calculated through the density functional theory in previous reports, which are much higher than those on nonpolar carbon surface,^18^, ^44^ revealing an inherent energetically favorable interaction between LiPSs and Ni_3_S_2_. These results confirm the superior adsorption and binding capabilities of S/CNF‐HC‐Ni_3_S_2_ for LiPSs, which are responsible for the phenomenal cycle stability and high utilization of sulfur.

Third, the S/CNF‐HC‐Ni_3_S_2_ cathode holds cooperative interfaces of “lithiophilic” heteroatom‐doped carbon and “sulfiphilic” Ni_3_S_2_, which can help to address the shuttle and kinetics issues synchronously by binding polysulfides and enhancing affinity to Li (e.g., Li^+^ ions and/or terminal Li in LiPSs) and transport of charge carriers. As confirmed by the spectroscopic characterization in Figure 3, the pristine S/CNF‐HC‐Ni_3_S_2_ electrode contains heteroatom‐doped carbon (e.g., C—S and C—O bonding), which is found to exhibit desirable lithiophilicity via Li—S or Li—O bonds.[qv: 33c,45] Meanwhile, Ni_3_S_2_ demonstrates favorable sulfiphilicity to bind the terminal S of LiPSs with exposed Ni sites via Ni—S bonds, which is supported by reported results for hybrid metal sulfide‐LiPS and other metal‐site‐containing host‐LiPSs systems.^14^, ^18^, ^46^ Such binary cooperative complementary with distinct chemisorptivity is conducive to enriching Li local concentration in the vicinity of cathode surface for promoting Li^+^‐transfer induced kinetics and also to immobilizing LiPSs on the heterogeneous surfaces for facilitating electrocatalytic redox conversion and restraining LiPSs shuttling. The cooperative interfaces of “lithiophilic” heteroatom‐doped carbon and “sulfiphilic” Ni_3_S_2_ for interaction with LiPSs are tentatively suggested by the XPS peak shifts of C 1s (C—S), S 2p, and Ni 2p_3/2_ and the changes of Li 1s and O 1s (O—Li) of S/CNF‐HC‐Ni_3_S_2_ cathode after 100 cycles.[qv: 33c] Further synchrotron‐based spectroscopic characterization and theoretical calculations will be employed for in‐depth understanding of the role of such cooperative interfaces in Li–S electrochemistry. The cooperative interfaces are proposed to play critical roles in the enhanced cycling stability (Figure 4) and accelerated redox kinetics as well as reduced charge transfer resistance (Figure 5) of S/CNF‐HC‐Ni_3_S_2_ beyond those of S/CNF‐HC. Fourth, the binder‐free freestanding S/CNF‐HC‐Ni_3_S_2_ electrode architecture containing electronically conductive Ni foam framework, HC and CNF layers (Figures 1 and 2) provides an efficient 3D electron pathway network, enabling fast electron transport to interfacial adsorbed LiPSs and Ni_3_S_2_ electrocatalyst for fast redox kinetics and remarkable rate capability. Moreover, Ni_3_S_2_ also has a fairly low resistivity (1.8 × 10^−5^ Ω cm at room temperature).^18^, ^47^ Such 3D interconnected continuous electron channels is benefited from the strong coupling of various components, rendering electrons to readily reach the LiPSs adsorption interfaces and electrocatalytic active sites and hence promoting the charge transfer and redox kinetics. In combination with the strong LiPSs binding capability and cooperative interfaces, the conductive network S/CNF‐HC‐Ni_3_S_2_ electrode ensures its accelerated redox kinetics and small *R*_ct_ (Figure 5g) and exceptional rate performance (Figure 4d).

Above all, the elaborate structural design of S/CNF‐HC‐Ni_3_S_2_ integrates all building blocks of Ni foam, sulfurized HC/CNF forest, Ni_3_S_2_ particles, and S with their respective functions into an ensemble, demonstrating a synergistic effect on the outstanding cathode performances in Li–S cells. Ni foam acts as a robust and conductive framework. The heteroatom‐doped HC layer with desired lithiophilicity and electronic conductivity serves as a primary reservoir for loading of active sulfur, helps to bind LiPSs and enables fast electron transport to interfacial adsorbed LiPSs and Ni_3_S_2_ sites. The sulfurized CNF forest with analogous lithiophilicity and electronic conductivity can increase the access to electrolyte, shorten the electron transport, facilitate the Li‐ion transport, and retard the LiPSs diffusion as a barrier layer. Sulfiphilic Ni_3_S_2_ acts as both a chemical anchor with strong chemical LiPSs binding capability for immobilizing soluble LiPSs at the interfacial sites and an efficient electrocatalyst with high catalytic activity for reducing the energy barriers and facilitating the kinetics of redox reactions. To highlight the structural merits of S/CNF‐HC‐Ni_3_S_2_, two control samples, bare Ni_3_S_2_/Ni and Ni foam coated by carbon microspheres layer with sulfur (S/CMP) electrodes together with the aforementioned S/CNF‐HC cathode were prepared by modified procedures for comparison. The Ni_3_S_2_/Ni electrode contributes to a limited capacity (4–6 mAh g^−1^) within the potential window of 1.7–3.0 V (Figure S19, Supporting Information). The S/CMP electrode without functional units of HC, CNF, and Ni_3_S_2_ contains crystalline bulk sulfur in the macropores of Ni foam and on the surface of CMP, exhibiting rapid decay of capacity and much lower coulombic efficiency (Figure S20, Supporting Information). The polysulfides shuttle resulted in the seriously contaminated separator and corroded Li anode with the dark yellow LiPSs solution when soaked the S/CMP electrode only after 30 cycles in the DME solvent (Figure 6c). In the absence of Ni_3_S_2_ as the chemical anchor and electrocatalyst, S/CNF‐HC cathode also shows poor cycling stability (Figure 4c) and inferior redox kinetics (Figure 5). These results indicate the severe shuttling of LiPSs with low sulfur utilization and sluggish redox kinetics in the cathodes without functional building blocks. By contrast, S/CNF‐HC‐Ni_3_S_2_ exhibits not only remarkable cycle stability and rate capability but also good structure stability. After 100 cycles, S/CNF‐HC‐Ni_3_S_2_ still maintains the texture (Figure 6b) and surface structure with intact CNFs and Ni_3_S_2_ which securely bind the lithium polysulfide and/or sulfide (Figure S21, Supporting Information) without any bulk sulfur species aggregated on the surface (Figure S22, Supporting Information), indicating little pulverization and volume changes in the electrode. Synergistically, all building blocks of S/CNF‐HC‐Ni_3_S_2_ promote the lithium ion coupled electron transfer for redox conversion and retention of LiPSs intermediates in the Li–S battery electrochemistry.

## 3 Conclusions

In summary, a bioinspired hierarchical electrode structure design is developed to integrate multiple functional units of Ni foam, HC, CNF forest, Ni_3_S_2_, and sulfur into an ensemble to obtain a versatile and high‐performance cathode (S/CNF‐HC‐Ni_3_S_2_) for Li–S batteries through a programmed fabrication approach. These building blocks have respective specialized functions. Overall, such integrated electrode demonstrates a synergy and thus provides a robust and effective 3D conductive network and cooperative interfaces to minimize the shuttle effect and enhance rate and cycling performances by increasing the density of adsorption sites, adsorption capability, electron/ion transfer and catalytic redox kinetics for the sulfur species during the discharge–charge process. Due to such unique structural design, the S/CNF‐HC‐Ni_3_S_2_ cathode delivers high reversible capacities of ≈850 mAh g^−1^ at 0.2 C after 100 cycles and ≈400 mAh g^−1^ at 5 C after 450 cycles. This work provides a promising cathode candidate and a novel programmed fabrication strategy for rational design of versatile electrodes for high‐energy Li–S batteries.

## 4 Experimental Section

*Surface Modification of Ni Foam*: A piece of Ni form was first punched into circular disks with a diameter of ≈10 mm and then pressed under 1500 lb pressure for 2 min to keep the mechanical strength during the following process. Five Ni form disks were subsequently immersed into the 80 mL deionized (DI) water dissolving 0.45 g FeSO_4_ · 7H_2_O, 0.2 g urea, and 0.016 g sodium lauryl sulfate. The transparent yellow solution with Ni disks was then transferred into a Teflon‐lined stainless steel autoclave with a capacity of 100 mL for hydrothermal treatment at 100 °C for 12 h. The obtained Ni foam disks were collected and washed with DI water and absolute ethanol several times and then dried under vacuum at 80 °C. In this way, the Ni foams were coated with NiFe_2_O_4_ layers (Figures S1 and S2, Supporting Information).

*Synthesis of 3D Conductive S/CNF‐HC‐Ni_3_S_2_ Electrode*: The NiFe_2_O_4_ modified Ni foam disks arranged on a Ni foam slab shelving on a combustion boat (Figure S3, Supporting Information) were put into a quartz tube for CVD process. The toluene was used as the carbon source and carried by 5% H_2_/Ar at a flow rate of 0.1 L min^−1^. The reaction system was heated to 800 °C at a ramping rate of 5 °C min^−1^ and maintained at this temperature for 3 h to enable the growth of carbon nanofibers and host carbon layers (CNF‐HC) over premodified Ni foam disks. After the CVD process, each Ni foam disk was mixed with ≈50 mg sulfur powder and sealed in a separate vial under the protection of Ar gas and then heated at 300 °C for 1 h to create the chemical anchors mainly composed of Ni_3_S_2_ and sulfurized carbon. Then a certain amount of sulfur powder (≈10 mg) was mixed with each Ni foam disk, which was heated at 155 °C in Ar for 12 h to infiltrate sulfur into host carbon layers. Finally, the disk was rapidly rinsed by CS_2_ to remove possible bulk sulfur on the surface and then the monolithic S/CNF‐HC‐Ni_3_S_2_ electrode was obtained. The sulfur loading for each disk was controlled to ≈2 mg cm^−2^ unless stated otherwise.

*Synthesis of 3D Conductive S/CNF‐HC Electrode*: The control sample, S/CNF‐HC, was synthesized by a similar method except for the absence of thermal sulfurization treatment at 300 °C. After the CVD growth of CNF‐HC layers on Ni foam disks, a certain amount of sulfur powder (≈10 mg) was mixed with each disk, which was heated at 155 °C in Ar for 12 h to infiltrate sulfur into host carbon layers. Finally, the disk was rapidly rinsed by CS_2_ to remove possible bulk sulfur on the surface. The S/CNF‐HC electrode has the similar architecture yet without Ni_3_S_2_ component.

*Synthesis of 3D Conductive S/CMP Electrode*: As a comparison, pristine Ni foam disks without hydrothermal pretreatment were also used in the similar CVD process to enable the growth of CMPs layers over their surfaces. In the absence of formation of chemical anchors at 300 °C, the same amount of sulfur powder was mixed with CMP‐modified Ni foam disk, which was heated at 155 °C in Ar for 12 h to infiltrate sulfur into CMP layers. Finally, the disk was also rinsed by CS_2_ and the self‐supported S/CMP electrode was obtained.

*Synthesis of Ni_3_S_2_/Ni Electrode*: The Ni_3_S_2_/Ni electrode was prepared by directly mixing the Ni foam disks and sulfur powder for the thermal sulfurization at 300 °C for 1 h in Ar.

*Materials Characterization*: XRD phase structures of the samples were characterized by PANalytical X'Pert Pro X‐ray diffractometer at 45 kV and 40 mA using Cu Kα radiation. The mass ratio of sulfur on each electrode was estimated by TGA (SDT Q600). The microstructure and morphology of the samples were observed by an SEM (Hitachi S‐4700) equipped with EDX spectroscopy. The surface chemical states were characterized by Digilab FTS 7000/UMA 600 FTIR spectroscopy, Raman spectroscopy (Renishaw InVia, excited by 532 nm laser), and XPS (PHI VersaProbe 5000, energy range: 0–1486.6 eV binding energy with Al Kα source). To confirm the role of chemical anchors, the S/CNF‐HC‐Ni_3_S_2_||Li cell was disassembled in the glovebox after cycles for various characterizations. The working electrodes were washed with DME to remove electrolyte residues and then dried at 60 °C in the vacuum oven prior to XPS characterization.

*Electrochemical Measurements: Li–S Cells Assembly and Measurement*: The coin cells were assembled using either self‐supported S/CNF‐HC‐Ni_3_S_2_, S/CNF‐HC or S/CMP disks as the working electrode, lithium metal foil (MTI Corporation) as the counter electrode, and porous polypropylene (Celgard 2400) as a separator. The liquid electrolyte was 1 m lithium bis(trfluoromethanesulfonyl) imide (LiTFSI) and 1% LiNO_3_ dissolved in dioxolane (DOL) and DME (1:1 v/v). The coin cells (CR2032) were fabricated in an argon‐filled glove box (moisture and oxygen levels less than 1 ppm). The electrochemical performance of the cells was tested by Arbin BT2143 32CH with the voltage range between 1.7 and 3.0 V versus Li^+^/Li. The rate performance of the corresponding cells was tested at various current densities from 0.2 to 5 C (1 C = 1000 mA g^−1^). The cyclic voltammetry (CV) measurements were conducted with the electrochemical workstation (Gamry Interface 5000E) at a scan rate of 0.1 mV s^−1^ within a voltage range of 1.7–3.0 V.

*Symmetrical Cells Assembly and Measurement*: The electrodes for symmetrical cells were fabricated without the presence of elemental sulfur. Either CNF‐HC or CNF‐HC‐Ni_3_S_2_ disks were used as identical working and counter electrodes. 30 µL electrolytes containing 1 m Li_2_S_6_ dissolved in DOL/DME (1:1, v/v) was injected into each coin cell. The dark brown Li_2_S_6_ electrolyte was prepared by mixing Li_2_S ad S into the solvent at a molar ratio of 1:5 under stirring at 60 °C for 12 h in Ar. CV measurements of the symmetrical cells were performed at scan rate of 10 mV s^−1^ within a voltage range from −1.2 to 1.2 V. EIS measurements were performed with the Gamry Interface 5000E at open‐circuit potential with sinusoidal potential excitation of 5 mV amplitude. The frequency range was from 1 MHz to 0.1 Hz.

## Conflict of Interest

The authors declare no conflict of interest.

## Supporting information

Supplementary

## References

[1] a) J. Zhang , Y. Shi , Y. Ding , L. Peng , W. Zhang , G. Yu , Adv. Energy Mater. 2017, 7, 1602876;

[2] a) W. Ai , W. Zhou , Z. Du , Y. Chen , Z. Sun , C. Wu , C. Zou , C. Li , W. Huang , T. Yu , Energy Storage Mater. 2017, 6, 112;

[3] a) N. Xu , T. Qian , X. Liu , J. Liu , Y. Chen , C. Yan , Nano Lett. 2017, 17, 538; 27977209 10.1021/acs.nanolett.6b04610

[4] a) L. Kong , X. Chen , B. Li , H. Peng , J. Huang , J. Xie , Q. Zhang , Adv. Mater. 2018, 30, 1705219; 10.1002/adma.201705219 29178490

[5] a) J. Wang , S. Chew , Z. Zhao , S. Ashraf , D. Wexler , J. Chen , S. Ng , S. Chou , H. Liu , Carbon 2008, 46, 229;

[6] a) S. Zhang , Front. Energy Res. 2013, 1, 10;

[7] a) H. S. Park , S. B. Han , D. H. Kwak , G. H. Lee , I. Choi , D. H. Kim , K. B. Ma , M. C. Kim , H. J. Kwon , K. W. Park , ChemSusChem 2017, 10, 2202; 28296248 10.1002/cssc.201700147

[8] a) G. Hu , Z. Sun , C. Shi , R. Fang , J. Chen , P. Hou , C. Liu , H.‐M. Cheng , F. Li , Adv. Mater. 2017, 29, 1603835; 10.1002/adma.201603835 28036126

[9] a) S. Mei , C. J. Jafta , I. Lauermann , Q. Ran , M. Kärgell , M. Ballauff , Y. Lu , Adv. Funct. Mater. 27, 1701176;

[10] P. Zhao , H. Cui , J. Luan , Z. Guo , Y. Zhou , H. Xue , Mater. Lett. 2017, 186, 62.

[11] Y. Fan , Z. Yang , W. Hua , D. Liu , T. Tao , M. M. Rahman , W. Lei , S. Huang , Y. Chen , Adv. Energy Mater. 2017, 7, 1602380.

[12] a) X. Gu , C. Lai , J. Mater. Res. 2017, 1, 16;

[13] a) M. Li , J. Zhou , J. Zhou , C. Guo , Y. Han , Y. Zhu , G. Wang , Y. Qian , Mater. Res. Bull. 2017, 4, 509;

[14] Z. Yuan , H. Peng , T. Hou , J. Huang , C. Chen , D. Wang , X. Cheng , F. Wei , Q. Zhang , Nano Lett. 2016, 16, 519. 26713782 10.1021/acs.nanolett.5b04166

[15] a) L. Tan , X. Li , Z. Wang , H. Guo , J. Wang , ACS Appl. Mater. Interfaces 2018, 10, 3707; 29300086 10.1021/acsami.7b18645

[16] L. Luo , S. H. Chung , A. Manthiram , Adv. Energy Mater. 2018, 8, 1801014.

[17] a) S.‐H. Chung , L. Luo , A. Manthiram , ACS Energy Lett. 2018, 3, 568;

[18] Z. Li , S. Zhang , J. Zhang , M. Xu , R. Tatara , K. Dokko , M. Watanabe , ACS Appl. Mater. Interfaces 2017, 9, 38477. 29035508 10.1021/acsami.7b11065

[19] a) M. Li , X. Wu , J. Zeng , Z. Hou , S. Liao , Electrochim. Acta 2015, 182, 351;

[20] G. Partizan , B. Mansurov , B. Medyanova , A. Koshanova , M. Mansurova , B. Aliyev , X. Jiang , Eurasian Chem.‐Technol. J. 2016, 18, 283.

[21] S. Biniak , G. Szymański , J. Siedlewski , A. Świątkowski , Carbon 1997, 35, 1799.

[22] C. Luo , Y. Zhu , O. Borodin , T. Gao , X. Fan , Y. Xu , K. Xu , C. Wang , Adv. Funct. Mater. 2016, 26, 745.

[23] a) X. Yu , J. Xie , J. Yang , H. Huang , K. Wang , Z. Wen , J. Electroanal. Chem. 2004, 573, 121;

[24] X. Wang , J. Hu , Y. Su , J. Hao , F. Liu , S. Han , J. An , J. Lian , Chem. Eur. J. 2017, 23, 4128. 28133889 10.1002/chem.201605212

[25] a) J. Roman , W. Neri , A. Derré , P. Poulin , Carbon 2019, 145, 556;

[26] J. Kapitan , L. Hecht , P. Bouř , Phys. Chem. Chem. Phys. 2008, 10, 1003. 18259640 10.1039/b713965a

[27] a) C. Sandroff , D. Herschbach , J. Phys. Chem. 1982, 86, 3277;

[28] a) Z. Cheng , H. Abernathy , M. Liu , J. Phys. Chem. C 2007, 111, 17997;

[29] a) G. Li , J. Sun , W. Hou , S. Jiang , Y. Huang , J. Geng , Nat. Commun. 2016, 7, 10601; 26830732 10.1038/ncomms10601 PMC4740444

[30] N. A. Jarrah , F. Li , J. G. van Ommen , L. Lefferts , J. Mater. Chem. 2005, 15, 1946.

[31] Z. Li , Y. Jiang , L. Yuan , Z. Yi , C. Wu , Y. Liu , P. Strasser , Y. Huang , ACS Nano 2014, 8, 9295. 25144303 10.1021/nn503220h

[32] a) H. Wei , E. F. Rodriguez , A. S. Best , A. F. Hollenkamp , D. Chen , R. A. Caruso , Adv. Energy Mater. 2017, 7, 1601616;

[33] a) Z. Zhang , C. Zhao , S. Min , X. Qian , Electrochim. Acta 2014, 144, 100;

[34] X. Song , X. Li , Z. Bai , B. Yan , D. Li , X. Sun , Nano Energy 2016, 26, 533.

[35] W. Liu , J. Jiang , K. R. Yang , Y. Mi , P. Kumaravadivel , Y. Zhong , Q. Fan , Z. Weng , Z. Wu , J. J. Cha , Proc. Natl. Acad. Sci. USA 2017, 114, 3578. 28320950 10.1073/pnas.1620809114 PMC5389302

[36] a) Y. Li , K. K. Fu , C. Chen , W. Luo , T. Gao , S. Xu , J. Dai , G. Pastel , Y. Wang , B. Liu , ACS Nano 2017, 11, 4801; 28485923 10.1021/acsnano.7b01172

[37] a) Y. Wang , R. Zhang , Y.‐C. Pang , X. Chen , J. Lang , J. Xu , C. Xiao , H. Li , K. Xi , S. Ding , Energy Storage Mater. 2019, 16, 228;

[38] a) Y. You , Y. Ye , M. Wei , W. Sun , Q. Tang , J. Zhang , X. Chen , H. Li , J. Xu , Chem. Eng. J. 2019, 355, 671;

[39] a) J. Xu , D. Su , W. Zhang , W. Bao , G. Wang , J. Mater. Chem. A 2016, 4, 17381;

[40] a) X. Liang , C. Hart , Q. Pang , A. Garsuch , T. Weiss , L. F. Nazar , Nat. Commun. 2015, 6, 5682; 25562485 10.1038/ncomms6682

[41] L. Zhang , Z. Chen , N. Dongfang , M. Li , C. Diao , Q. Wu , X. Chi , P. Jiang , Z. Zhao , L. Dong , Adv. Energy Mater. 2018, 8, 1802431.

[42] Z. W. Seh , H. Wang , P.‐C. Hsu , Q. Zhang , W. Li , G. Zheng , H. Yao , Y. Cui , Energy Environ. Sci. 2014, 7, 672.

[43] a) S. Yuan , J. L. Bao , L. Wang , Y. Xia , D. G. Truhlar , Y. Wang , Adv. Energy Mater. 2016, 6, 1501733;

[44] G. Zhou , H. Tian , Y. Jin , X. Tao , B. Liu , R. Zhang , Z. W. Seh , D. Zhuo , Y. Liu , J. Sun , J. Zhao , C. Zu , D. S. Wu , Q. Zhang , Y. Cui , Proc. Natl. Acad. Sci. USA 2017, 114, 840. 28096362 10.1073/pnas.1615837114 PMC5293031

[45] a) Z. Xiao , L. Li , Y. Tang , Z. Cheng , H. Pan , D. Tian , R. Wang , Energy Storage Mater. 2018, 12, 252;

[46] a) R. Demir‐Cakan , M. Morcrette , F. Nouar , C. Davoisne , T. Devic , D. Gonbeau , R. Dominko , C. Serre , G. Férey , J.‐M. Tarascon , J. Am. Chem. Soc. 2011, 133, 16154; 21882857 10.1021/ja2062659

[47] P. Metcalf , P. Fanwick , Z. Kakol , J. Honig , J. Solid State Chem. 1993, 104, 81.

