# Supplementary material for: Programmed Design of a Lithium–Sulfur Battery Cathode by Integrating Functional Units
Source: Adv Sci (Weinh). 2019 Jul 19;6(17):1900711. doi: 10.1002/advs.201900711 (PMC6724479; doi:10.1002/advs.201900711)
Supplement: Supplementary file 1 — Supplementary [file ADVS-6-1900711-s001.pdf]

Copyright WILEY-VCH Verlag GmbH & Co. KGaA, 69469 Weinheim, Germany, 2019.

ADVANCED SCIENCE
Open Access

Supporting Information

for *Adv. Sci.,* DOI: 10.1002/advs.201900711

Programmed Design of a Lithium–Sulfur Battery Cathode by Integrating Functional Units

*Zhipeng Zeng, Wei Li, Qiang Wang, and Xingbo Liu**

Supporting Information

Programmed Design of a Li-S Battery Cathode by Integrating Functional Units

Zhipeng Zeng[1], *Wei Li*[1], *Qiang Wang*[2, 3], *Xingbo Liu*[1,]*

[1]Department of Mechanical & Aerospace Engineering, West Virginia University, Morgantown, WV 26506, USA

[2]Department of Physics and Astronomy, West Virginia University, Morgantown, WV 26506, USA

[3]Shared Research Facilities, West Virginia University, Morgantown, WV 26506, USA
* Corresponding author. E-mail address: xingbo.liu@mail.wvu.edu (X. Liu).

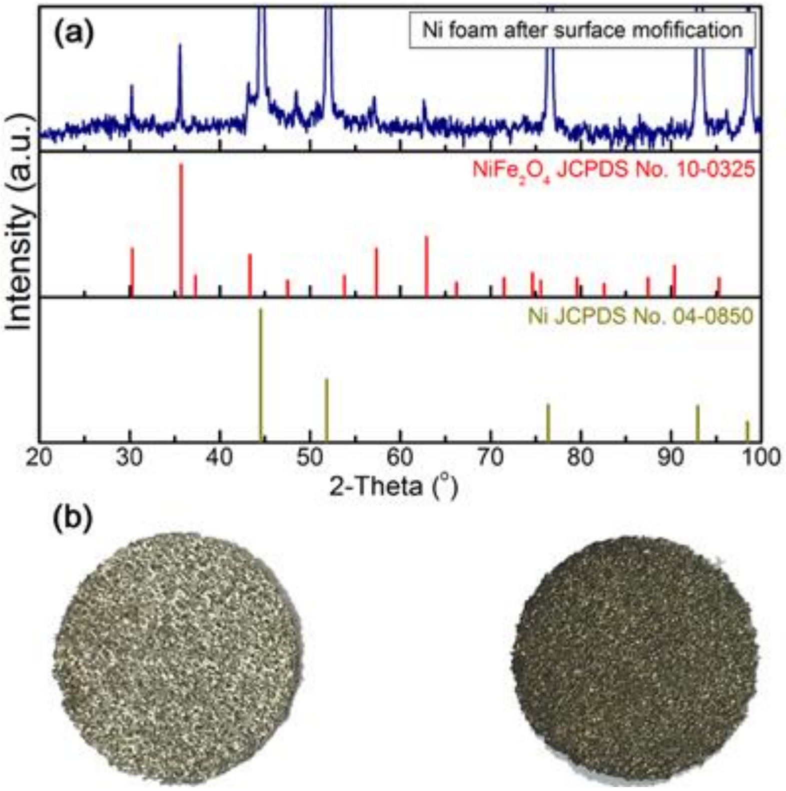

Figure S1 (a) XRD pattern of the $NiFe_2O_4$-modified Ni foam after the hydrothermal process and standard XRD cards of $NiFe_2O_4$ and Ni. (b) Photographs of Ni foam discs before (left) and after (right) the hydrothermal surface modification treatment.

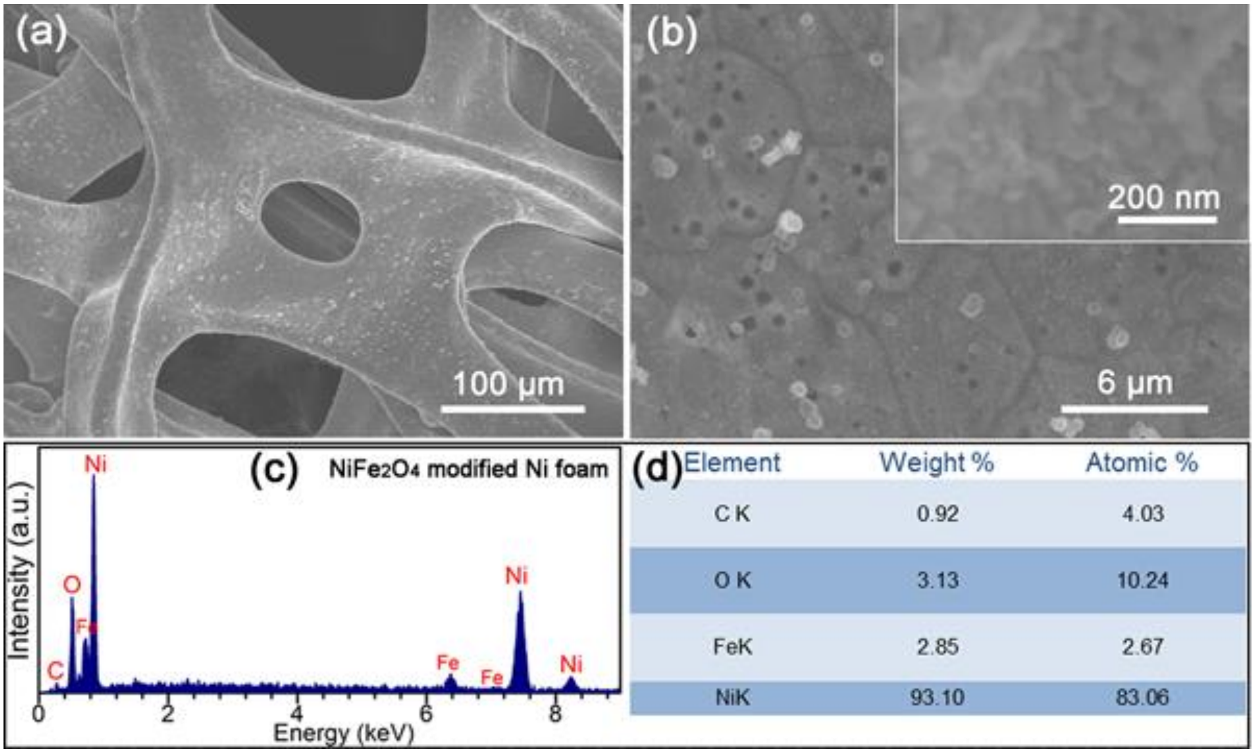

Figure S2 SEM images (a, b), EDX spectrum (c) and quantitative analysis (d) of $NiFe_2O_4$-modified Ni foam. Compared with the fresh Ni foam with a smooth surface (Figure S6), lots of nanoparticles can be clearly observed on the surface ligament of Ni foam, as seen in Figure S2b. The corresponding high-magnification image reveals that a very thin metal oxide layer can be formed on the Ni foam surface. The EDX results in Figure S2c and d further confirm the existence of Ni, Fe, and O.

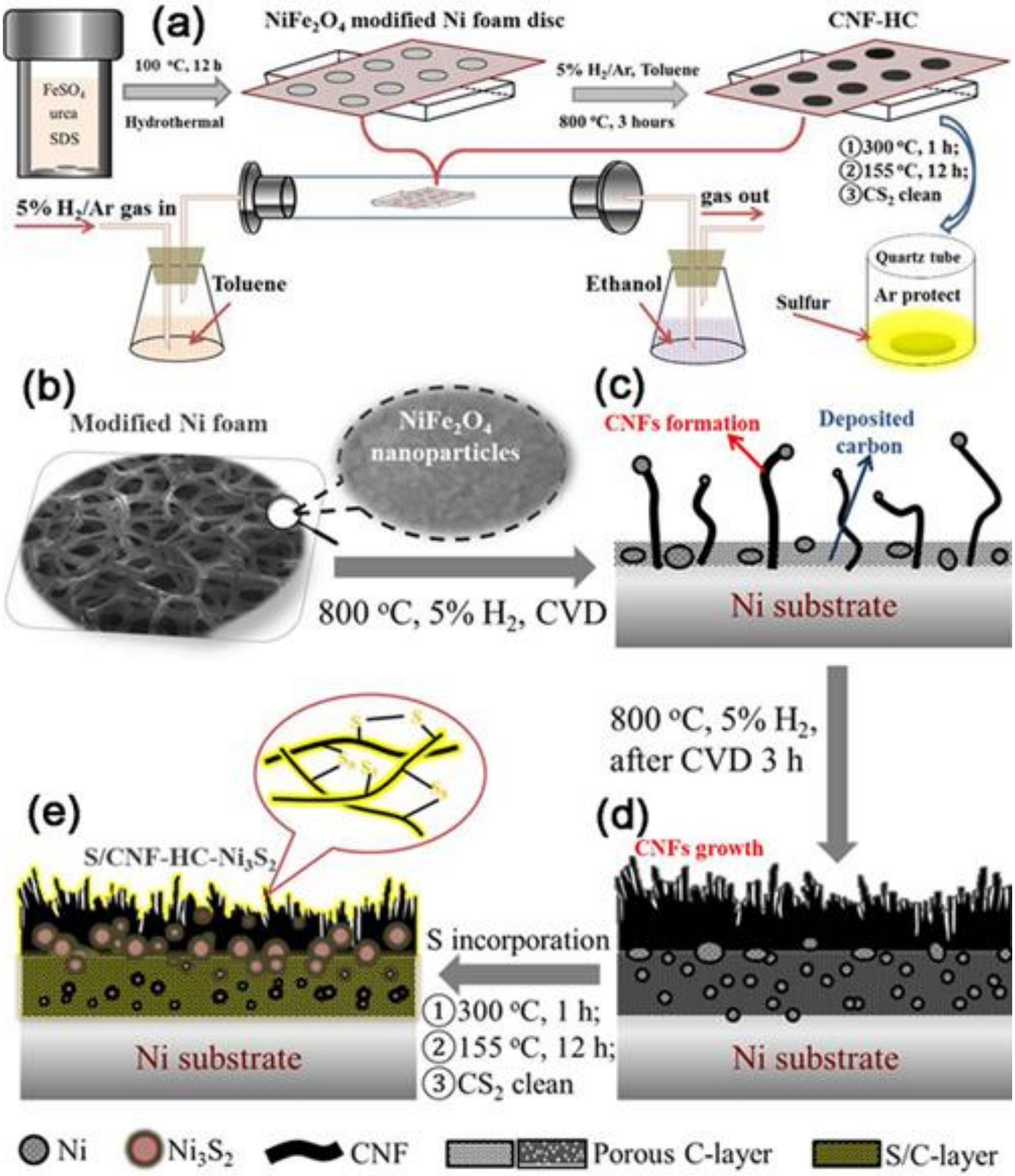

Figure S3 Schematic illustrations of the whole programmed fabrication process for synthesizing S/CNF-HC-Ni_3S_2.

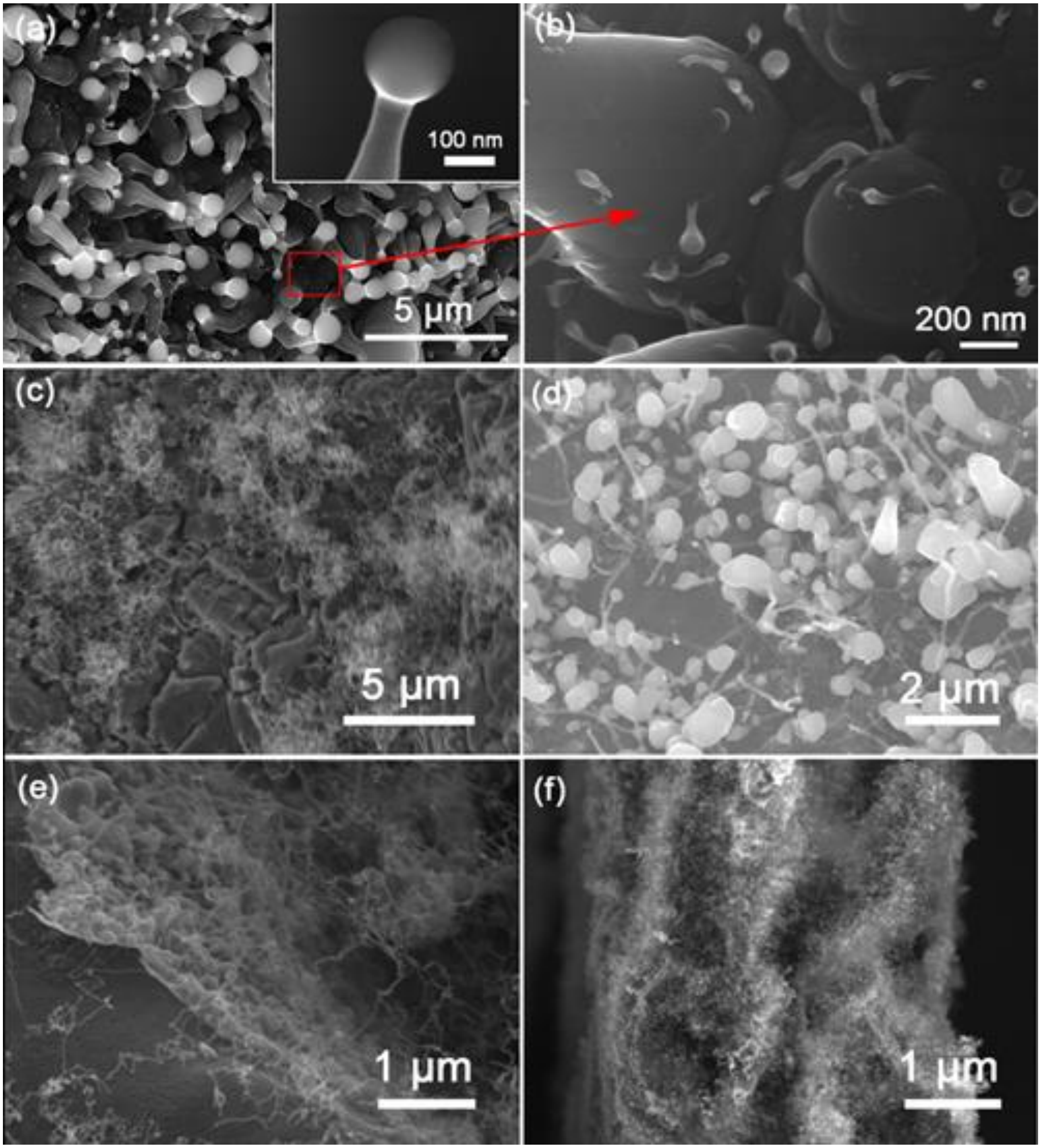

Figure S4 SEM images for the evolution of carbon nanofibers growing on the surface of the hydrothermally treated Ni foam at different stages: (a, b) early stage, (c, d) middle stage and (e, f) late stage.

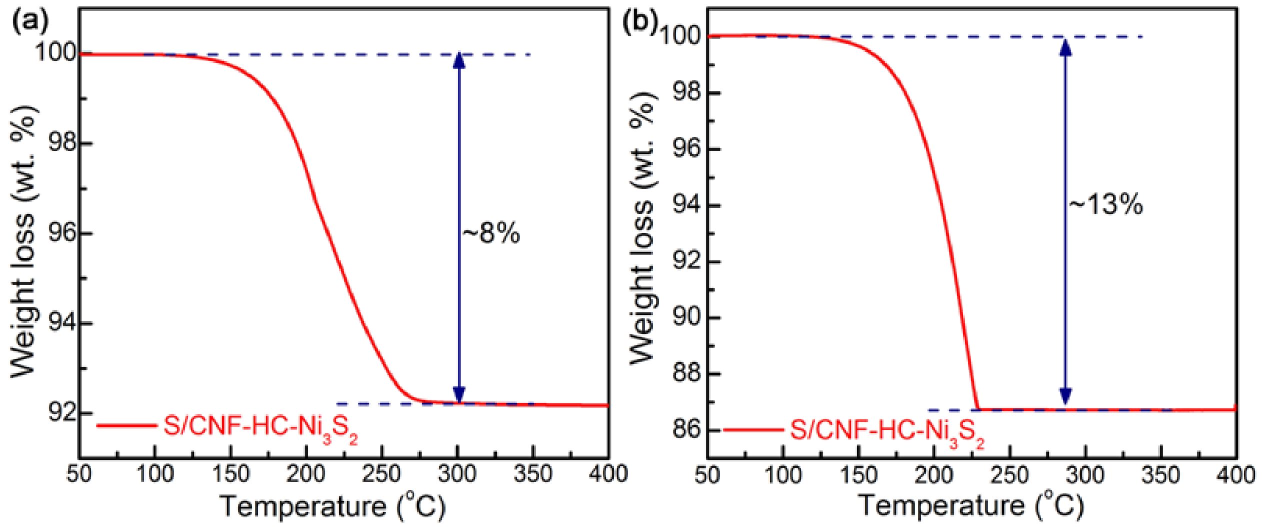

Figure S5 TG curves of the S/CNF-HC-Ni_3S_2 electrodes with sulfur mass ratio of 8 wt. % for No. 4 (a) and 13 wt. % for No. 8 electrodes (b) recorded under a N_2 atmosphere.

Table S1 Mass changes of electrodes after different treatment (*unit: mg*).

**Sample No.**	**Ni foam**	**CNF-HC**	**CNF-HC-Ni_3S_2**	**S/CNF-HC-Ni_3S_2**	**Loading ($mg\ cm^{-2}$)**	**S (wt. %)**
1	25.21	26.16	27.77	29.35	1.58	5.4%
2	25.24	26.71	27.89	29.36	1.84	5.0%
3	24.26	25.56	27.88	29.62	2.18	5.8%
4	24.17	25.83	28.12	30.32	2.75	7.3%
5	26.03	27.23	29.31	31.54	2.79	7%
6	25.36	26.14	29.66	33.13	4.34	10.5%
7	24.53	25.62	27.68	31.17	4.36	11.2%
8	25.44	26.82	28.87	32.83	4.95	12%

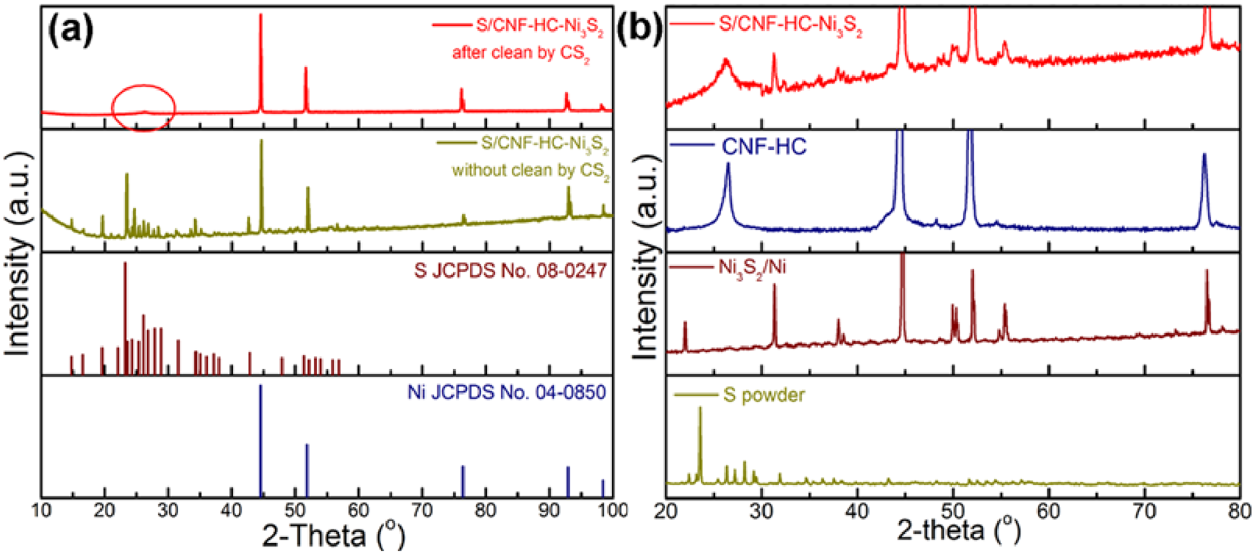

Figure S6 (a) XRD patterns of S/CNF-HC-Ni_3S_2 electrodes with and without CS_2 rinsing treatment, (b) detailed comparison for the XRD patterns of different electrodes. For the sample without further cleaning process (dark yellow curve), the dominant peaks are ascribed to the orthorhombic sulfur (JCPSD No. 08-0247) and Ni substrate (JCPDS No. 04-0850), indicating that a large amount of elemental sulfur still remained on the surface. However, the S/CNF-HC-Ni_3S_2 electrode after further cleaning by CS_2 (red curves) only shows the Ni diffraction peaks without any obvious peaks corresponding to the elemental sulfur. Notably, a diffraction hump can be observed in the marked zone, which should result from the carbon layers deposited on the surface of Ni foam. The typical peaks corresponding to graphitic carbon and Ni substrate can be identified in Figure S6b for CNF-HC. After sulfurization process, Ni_3S_2 nanoparticles can be formed and uniformly distribute in the carbon matrix, as the major peaks on the surface of S/CNF-HC-Ni_3S_2 electrode coincide with the Ni_3S_2/Ni electrode.

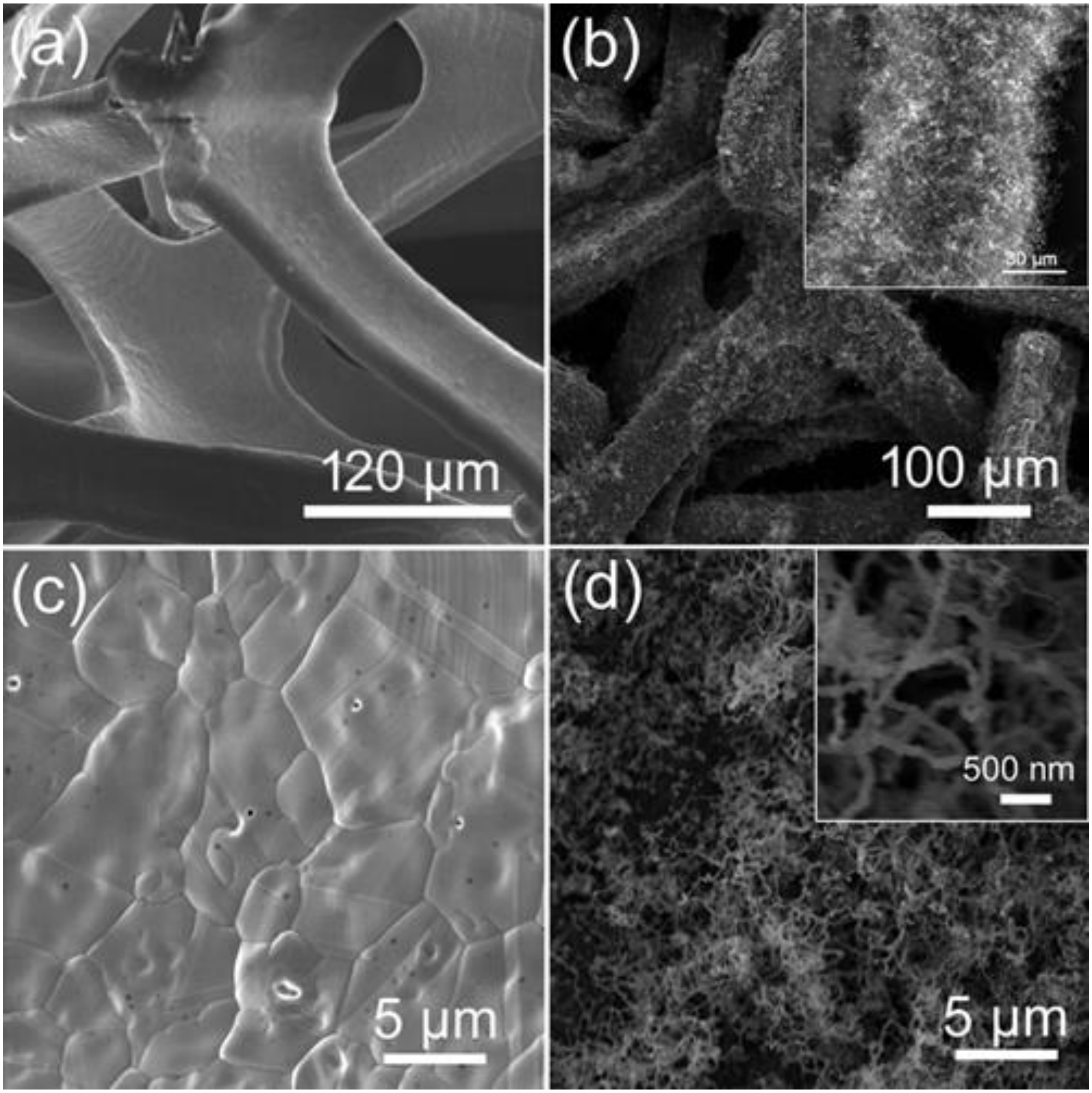

Figure S7 SEM images of fresh Ni foam at low (a) and high (c) magnification, and the SEM images for hydrothermally treated Ni foam after CVD process (b, d) with the flourishing growth of carbon nanofibers (CNFs) on the surface.

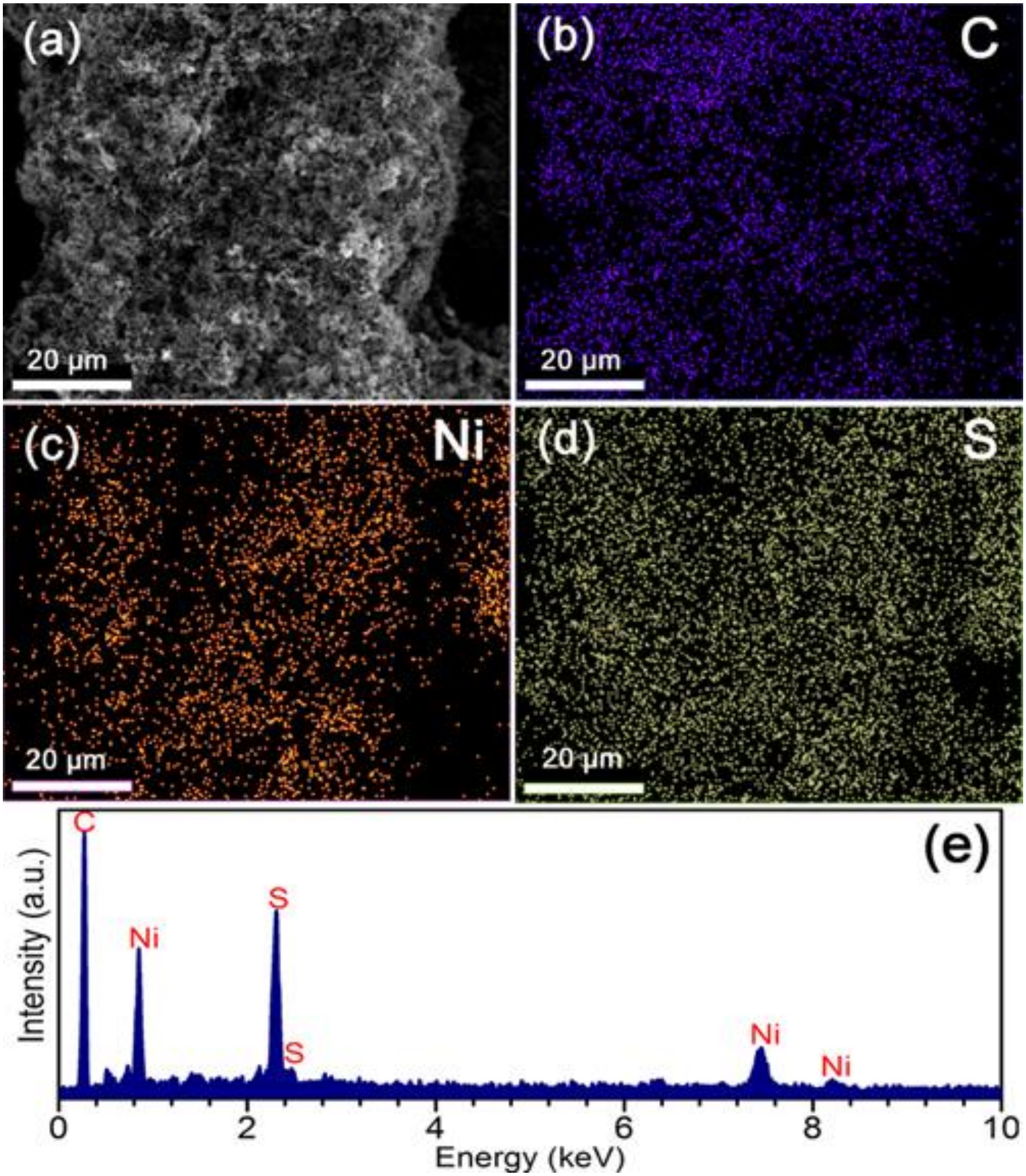

Figure S8 SEM image of pristine S/CNF-HC-Ni_3S_2 and corresponding elemental maps of C (b), Ni (c) and S (d) and its EDX spectrum.

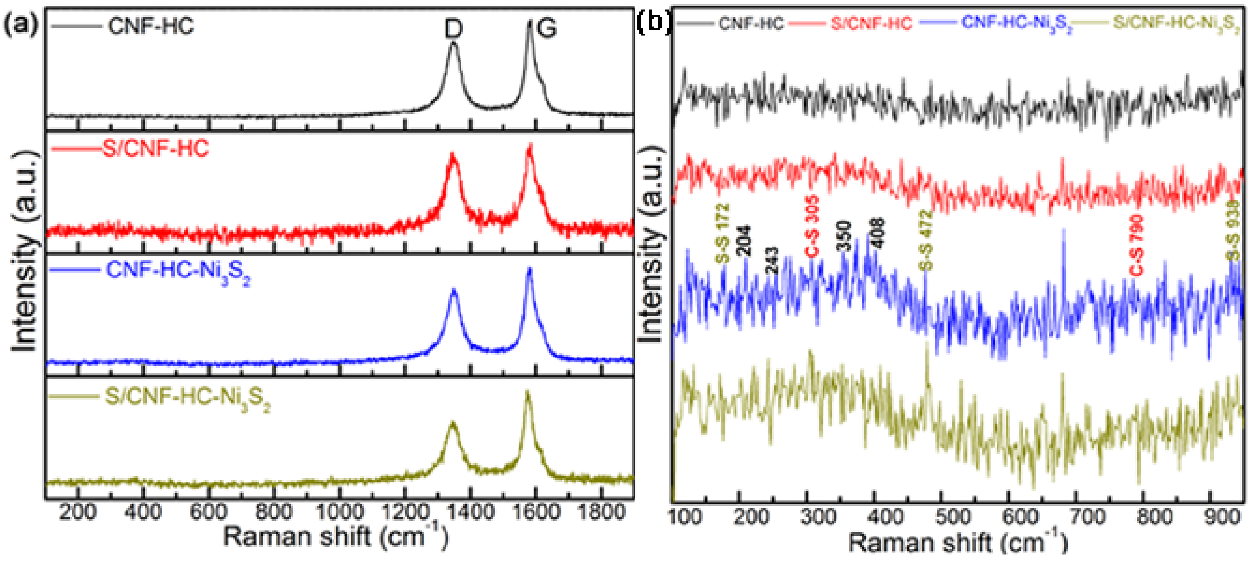

Figure S9 Raman spectrum of different electrodes and the detailed information enlarge capability of S/CNF-HC-Ni_3S_2 (b). By comparing the Raman curves for different components, it is clear that the sulfur diffusion process cannot introduce sufficient chemcial anchors into the electrode. However, after sulfurization process, Ni_3S_2 nanoparticles and sulfurized carbon can be created, as indicated in Figure S9b. Two peaks of 790 and 938 cm^{-1} correspond to C-S and S-S vibrations, respectively. The peaks at 204, 243, 350, 408 cm^{-1}, can be assigned to the vibrational modes of nickel sulfides. In addition, the characteristic peaks of C-S (305 cm^{-1}) and S-S (172 and 472 cm^{-1}) can also be identified.

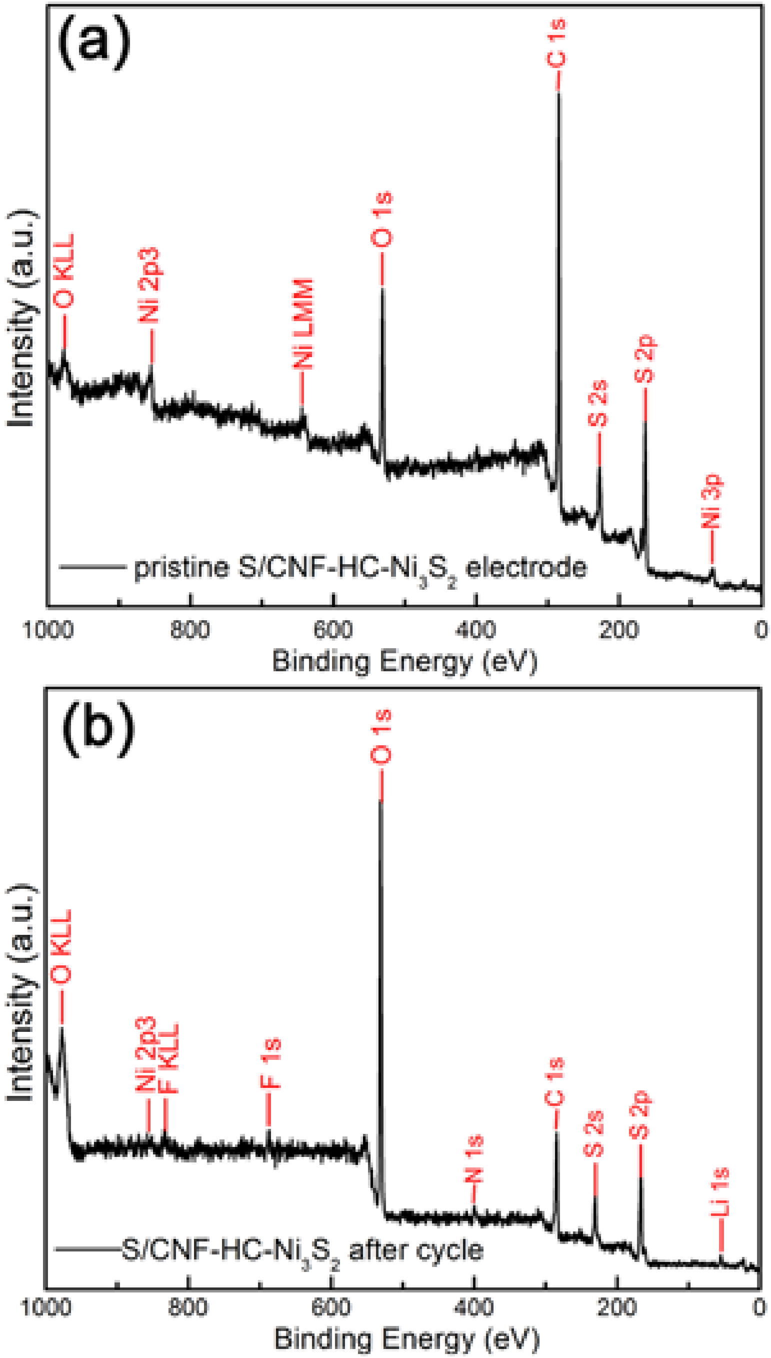

Figure S10 XPS survey scans of S/CNF-HC-Ni_3S_2 electrodes before (a) and after (b) 100 cycles.

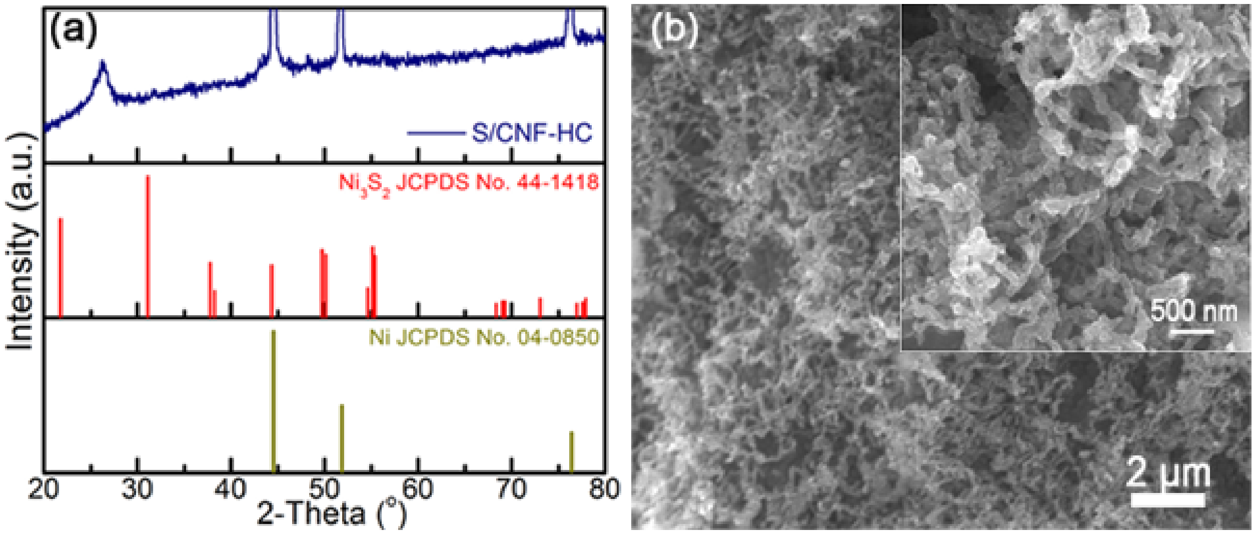

Figure S11 (a) XRD pattern and (b) SEM image of S/CNF-HC electrode. Basically, the morphology of S/CNF-HC electrode seems no significant change compared with that of S/CNF-HC-Ni_3S_2 electrode. However, no peaks corresponding to Ni_3S_2 can be observed from the XRD pattern, indicating that Ni_3S_2 was not formed without the heat treatment at 300 ^oC.

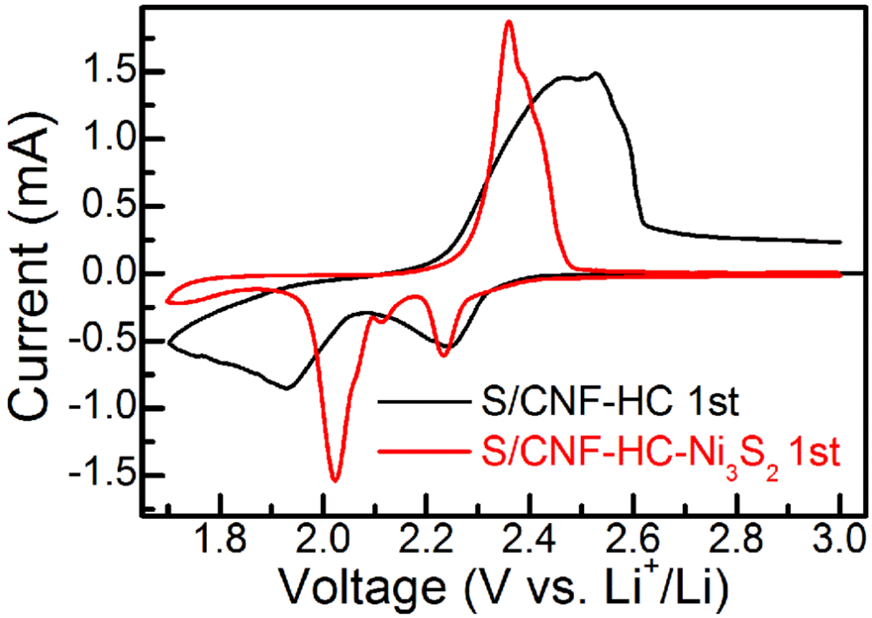

Figure S12 The first CV curves of S/CNF-HC-Ni_3S_2 and S/CNF-HC electrode. The sharp and narrow cathodic and anodic peaks of S/CNF-HC-Ni_3S_2 electrode indicate the fast redox kinetics and less polarization.

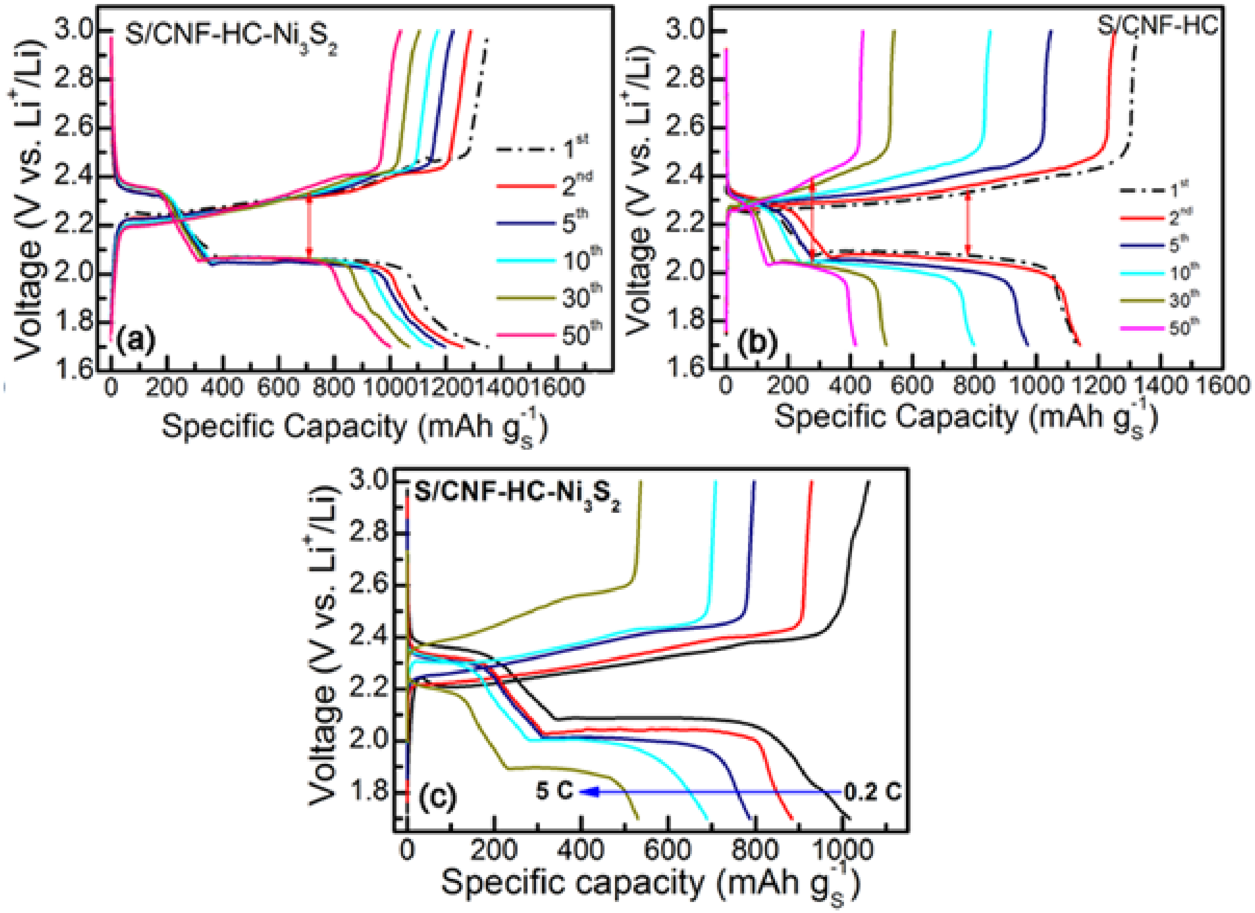

Figure S13 The charge/discharge curves of S/CNF-HC-Ni_3S_2 (a) and S/CNF-HC electrode (b). Galvanostatic discharge-charge profiles of S/CNF-HC-Ni_3S_2 electrode at various current densities (c).

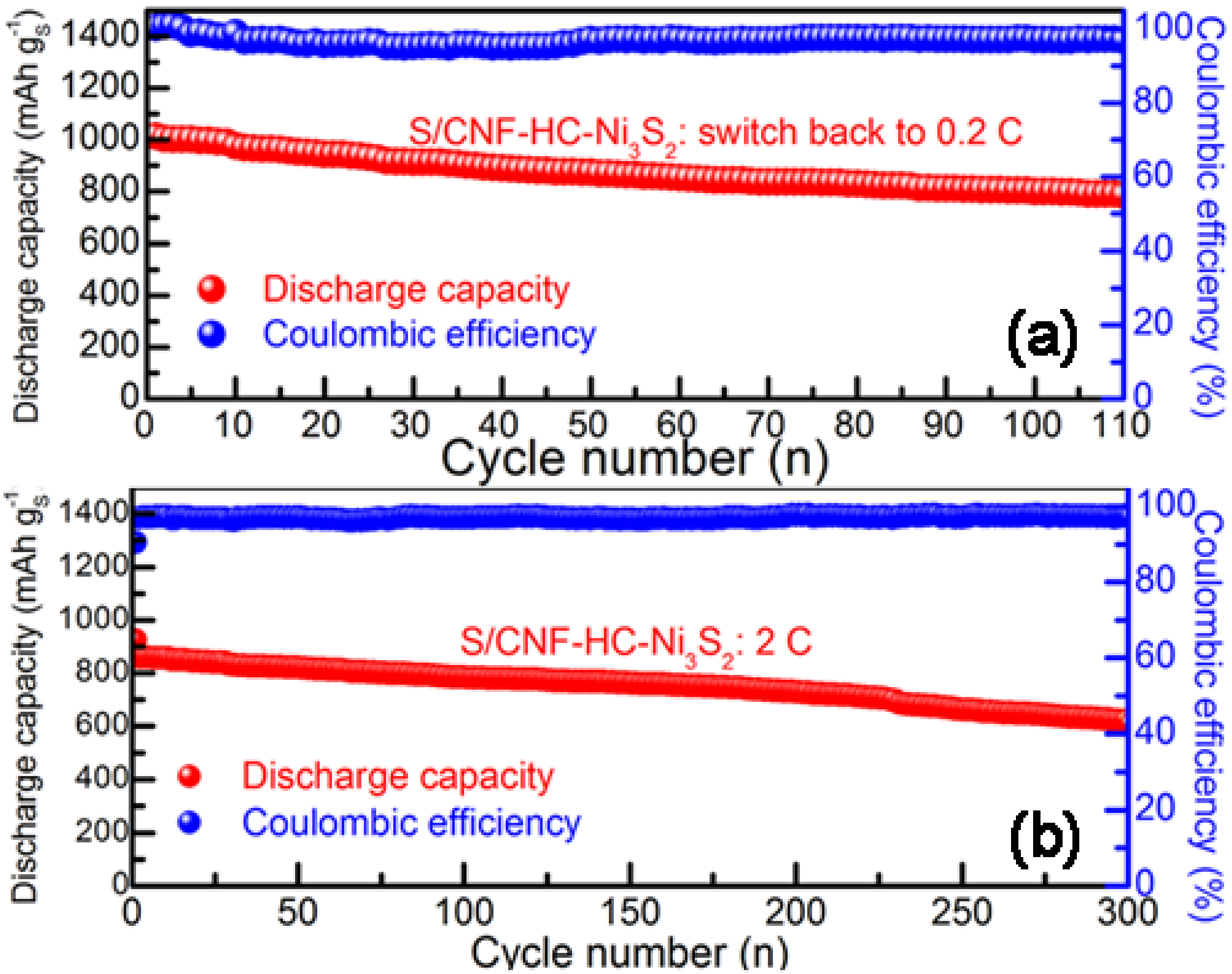

Figure S14 Cycle performance of S/CNF-HC-Ni_3S_2 electrodes on return to 0.2 C for 100 cycles (a), and 2 C for 300 cycles (b).

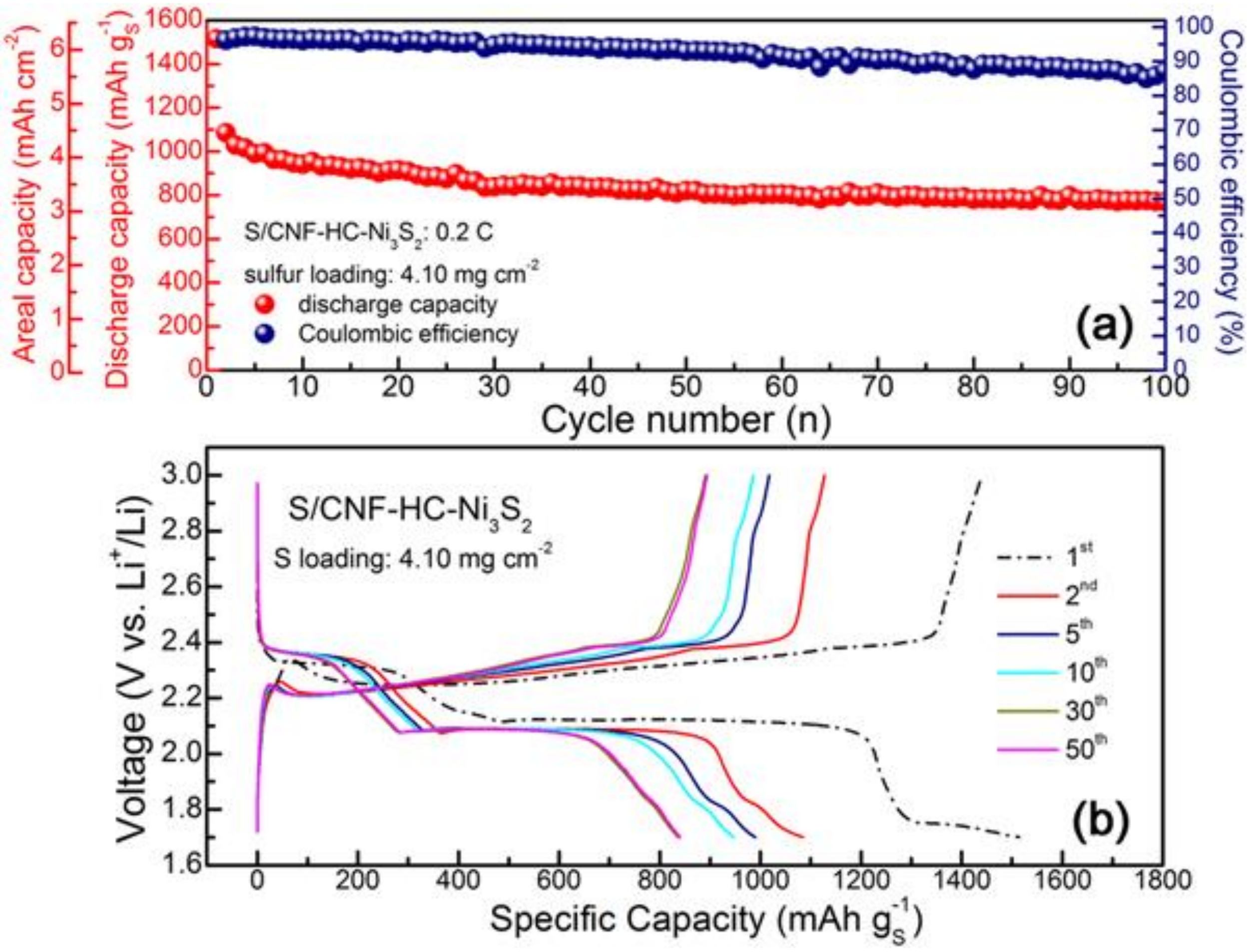

Figure S15 Cycle performance of S/CNF-HC-Ni_3S_2 electrode with a relatively high loading tested at 0.2 C (a) and corresponding charge-discharge curves (b).

Table S2 The performance comparison of S/CNF-HC-Ni_3S_2 with reported cathodes.

Cathodic materials	Mass loading (mg cm^{-2})	Rate	Capacity (mAh g^{-1})	Areal capacity (mAh cm^{-2})	Reference
**S/CNF-HC-Ni_3S_2**	**2.31**<br>**2.13**<br>**2.20**<br>**4.11**	**0.2 A g^{-1}**<br>**2 A g^{-1}**<br>**5 A g^{-1}**<br>**0.2 A g^{-1}**	**850 (100)**<br>**620 (300)**<br>**~400 (450)**<br>**760 (100)**	**1.97**<br>**1.32**<br>**0.88**<br>**3.12**	**This work**
**S/NiS@C-HS**	1.0	0.2 C	718 (200)	0.72	[S1]
**TiN-S**	4.6	0.77 mA cm^{-2}	685 (50)	3.15	[S2]
**S/IKB**	4.7	1.57 mA cm^{-2}	800 (90)	3.76	[S3]
**S_{2-4}/UMC**	4.2	2 mA cm^{-2}	580 (60)	2.44	[S4]
**S/Co_9O_8**	4.5	0.05 C	500 (150)	2.25	[S5]
**S/G-DBD**	1.6	0.2 C	754 (200)	1.21	[S6]
**Ni/Ni_3S_2/S**	4.0	1 mA cm^{-2}	654 (80)	2.62	[S7]
**S@NiCo-DH@RC**	1.5	0.2 C	972 (250)	1.46	[S8]
**MgB_2-S60**	2.0	0.5 C	~800 (200)	1.6	[S9]
**S/PPA**	1.5	0.5 C	763 (100)	1.14	[S10]
**Li_2S-$CoSe_2$/G**	4.35	0.5 C	832 (100)	3.62	[S11]
**TiN-C65**	0.6	0.2 C	680 (100)	0.41	[S12]
**PRC/Ni/S**	4.0	0.2 C	564 (300)	2.26	[S13]
**S/CNT/PrNP**	3.8	0.2 C	685 (200)	2.6	[S14]
**S@Ni/Fe LDH**	2.3	0.2 C	725 (200)	1.64	[S15]
**CNF sheets/S**	2.4	0.33 C	683 (500)	1.64	[S16]
**S/C with NCM**	4.0	0.5 C	~550 (100)	2.2	[S17]
**S/CNT with PNG**	3.6	1.5 mA cm^{-2}	688 (400)	2.48	[S18]
**C@TiN-S**	4.2	0.2 C	820 (150)	3.44	[S19]
**MoS_2/rGO/S**	0.85	0.5 C	680 (200)	0.58	[S20]

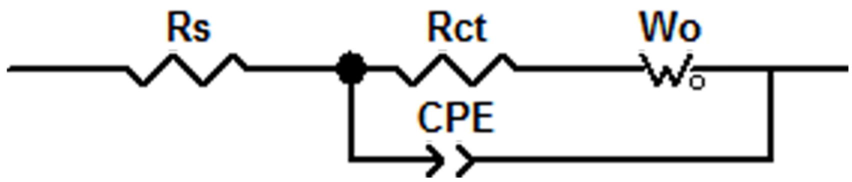

Cathode matrix	R_s (Ω cm^2)	R_{ct} (Ω cm^2)	CPE-T (Ω^{-1} S^n)	CPE-P
**CNF-HC**	**28.8**	**2214**	**1.87×10^{-5}**	**0.80**
**CNF-HC-Ni_3S_2**	**14.4**	**168**	**6.9×10^{-4}**	**0.71**

Figure S16 Equivalent circuit used for fitting the EIS spectra of symmetrical cells in Figure 5g and the corresponding fitting results.

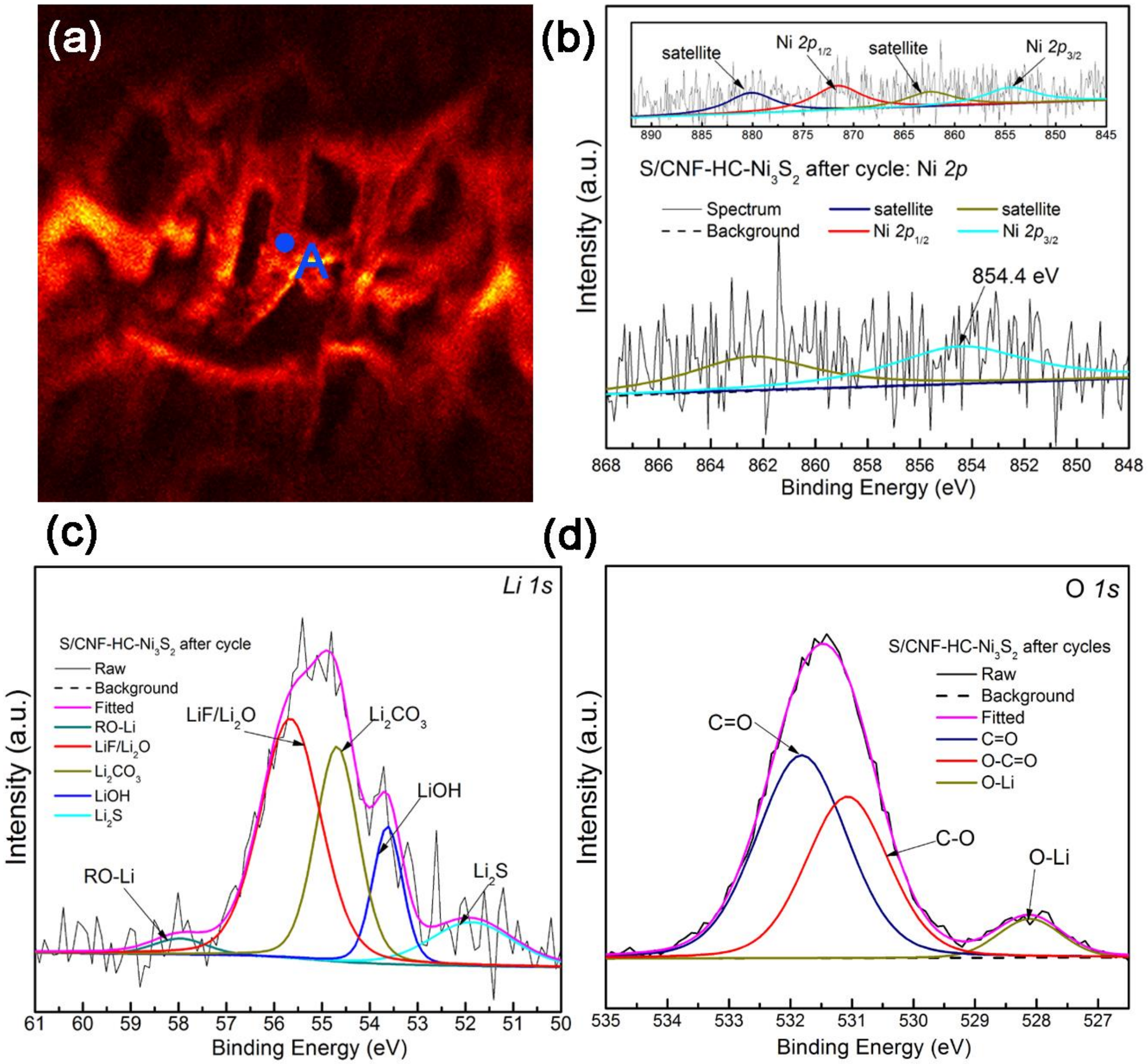

Figure S17 (a) XPS image with scan point A, high-resolution XPS spectra of Ni *2p* (b), Li *1s* (c), and O *1s* (d) of S/CNF-HC-Ni_3S_2 electrode after 100 cycles at the discharged state.

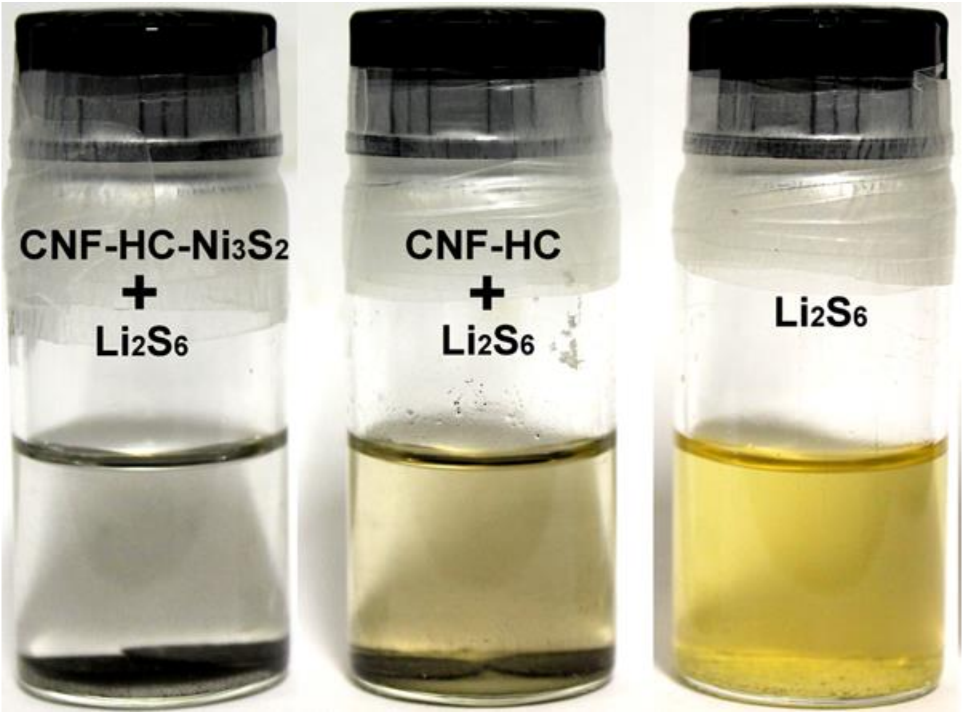

Figure S18 Adsorption ability test of CNF-HC-Ni_3S_2 and CNF-HC electrodes with the representative lithium polysulfides (0.1 M Li_2S_6).

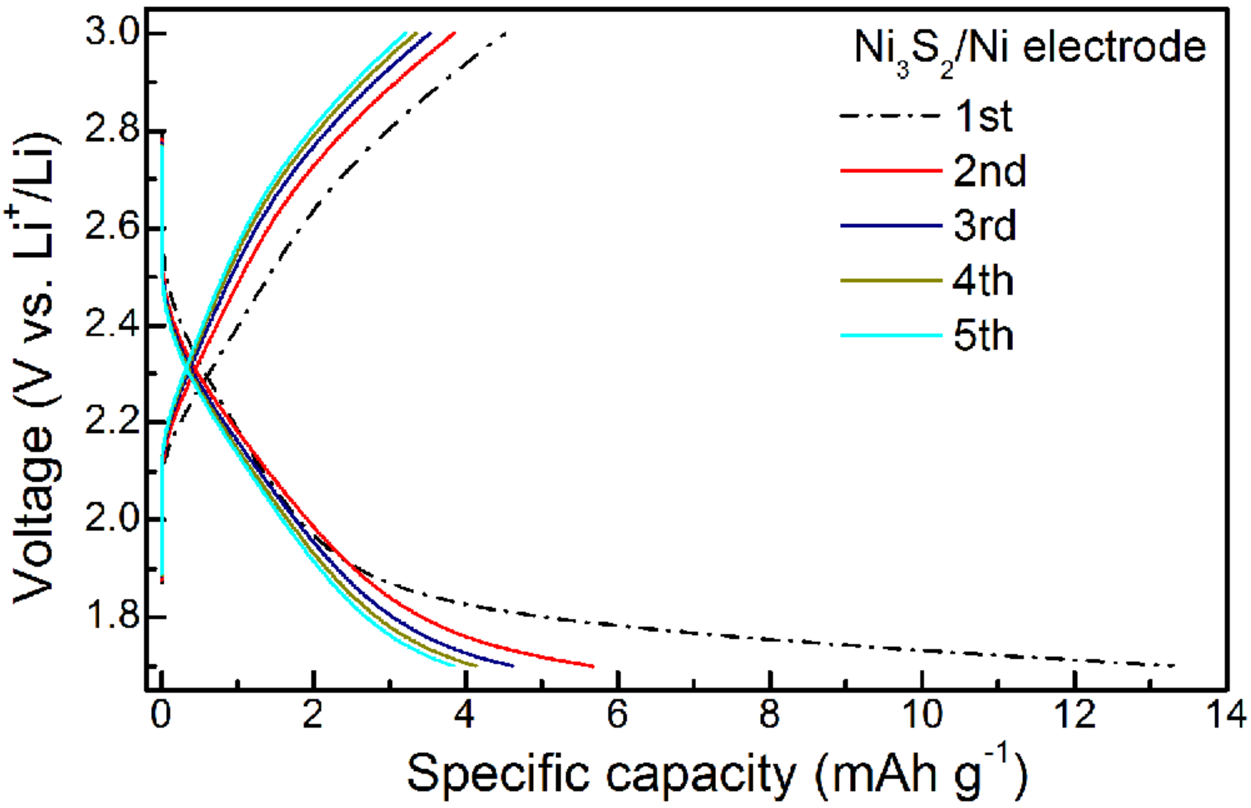

Figure S19 Charge/discharge profiles for the Ni_3S_2/Ni electrode. The Ni_3S_2/Ni electrodes were prepared by directly mixing the Ni foam discs and sulfur powder for the thermal sulfurization at 300 ^{o}C for 1 h in Ar. The Ni_3S_2/Ni electrode contributes to a limited capacity within the potential window of 1.7-3.0 V for testing Li-S cells.

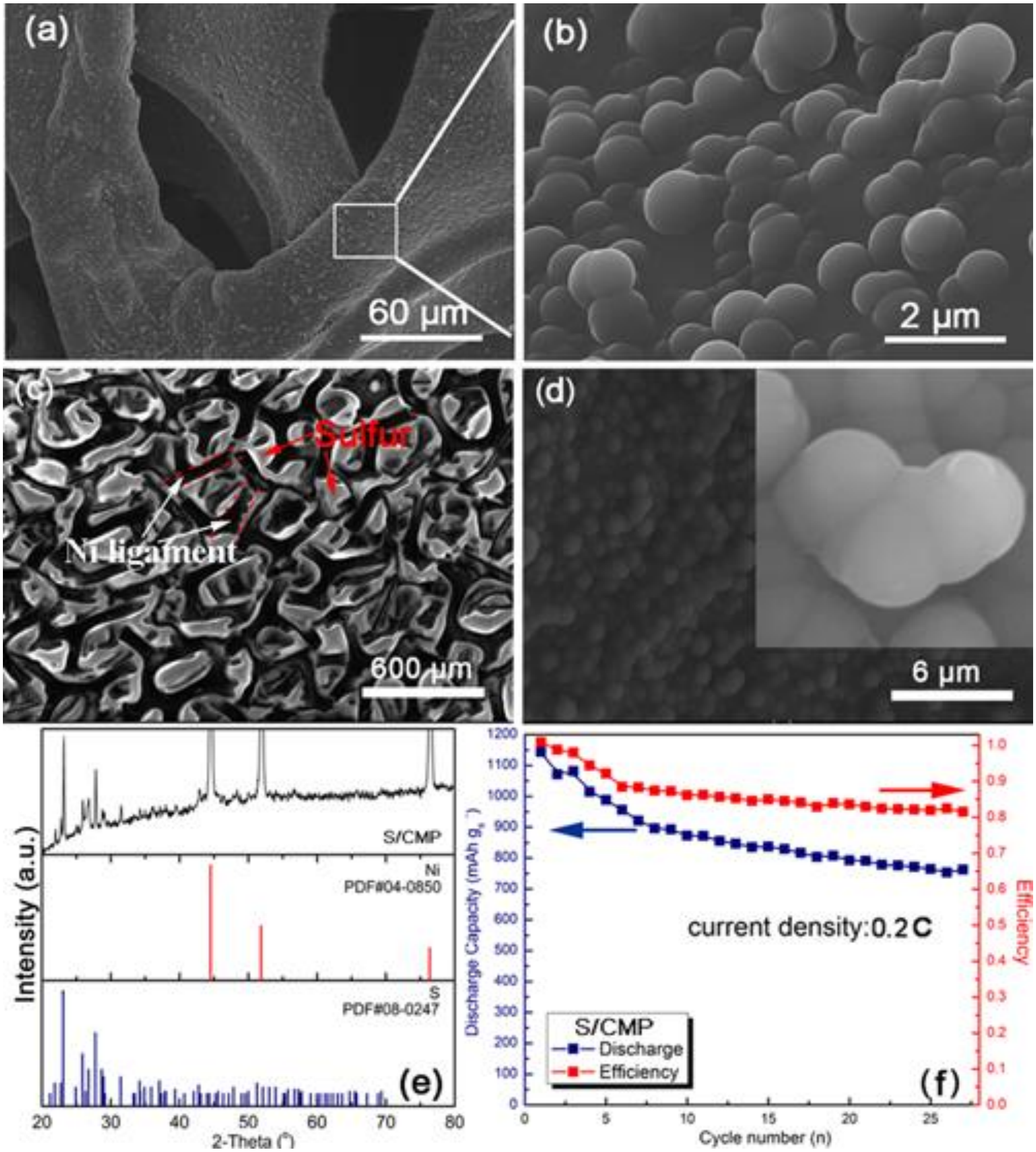

Figure S20 SEM images of carbon microspheres (CMPs) coated Ni foam at low (a) and high (b) magnification. SEM images of S/CMP electrode at low (c) and high (d) magnification. (e) XRD pattern of pristine S/CMP electrode and the corresponding cycle performance at 0.2 C (f).

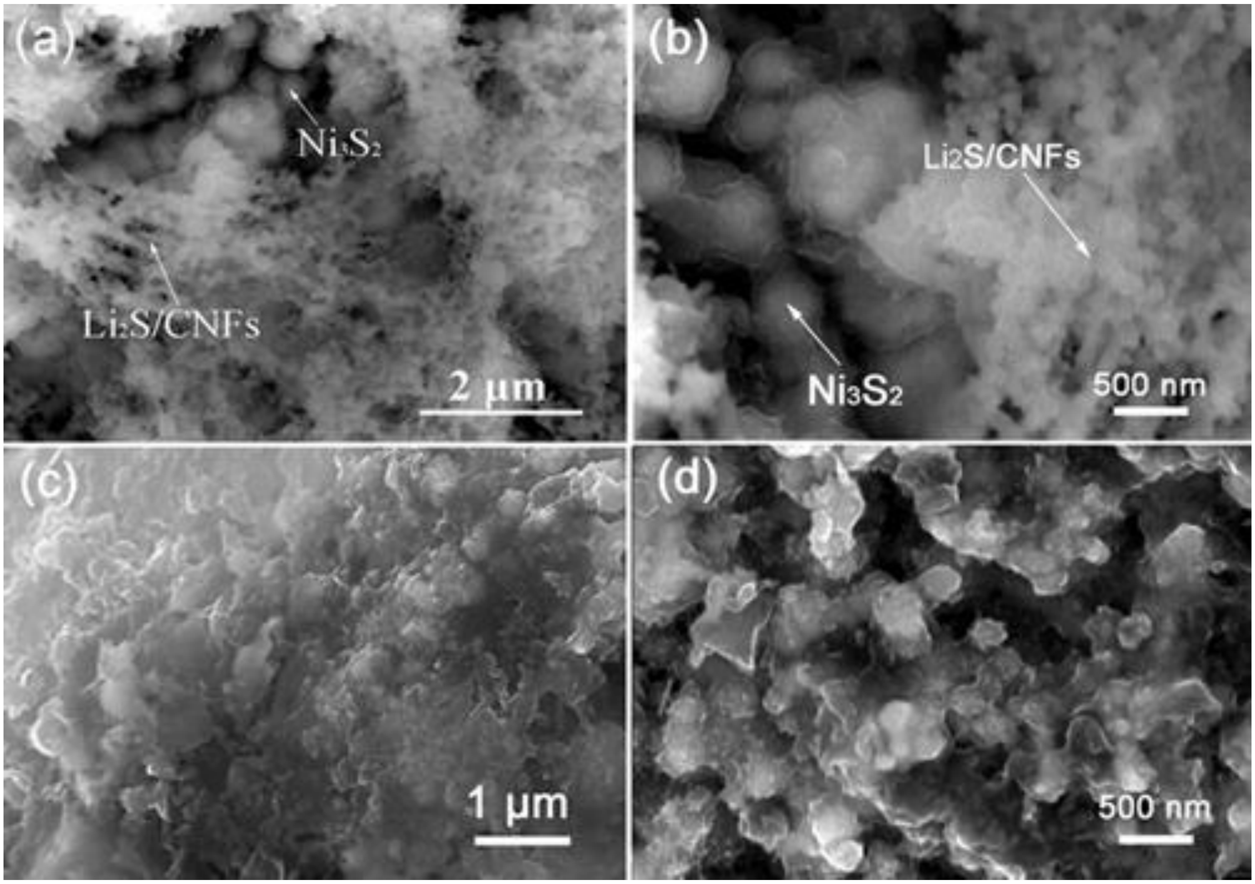

Figure S21 SEM images of the cycled electrodes of S/CNF-HC-Ni_3S_2 (a-b) and S/CMP (c-d) after 100 cycles.

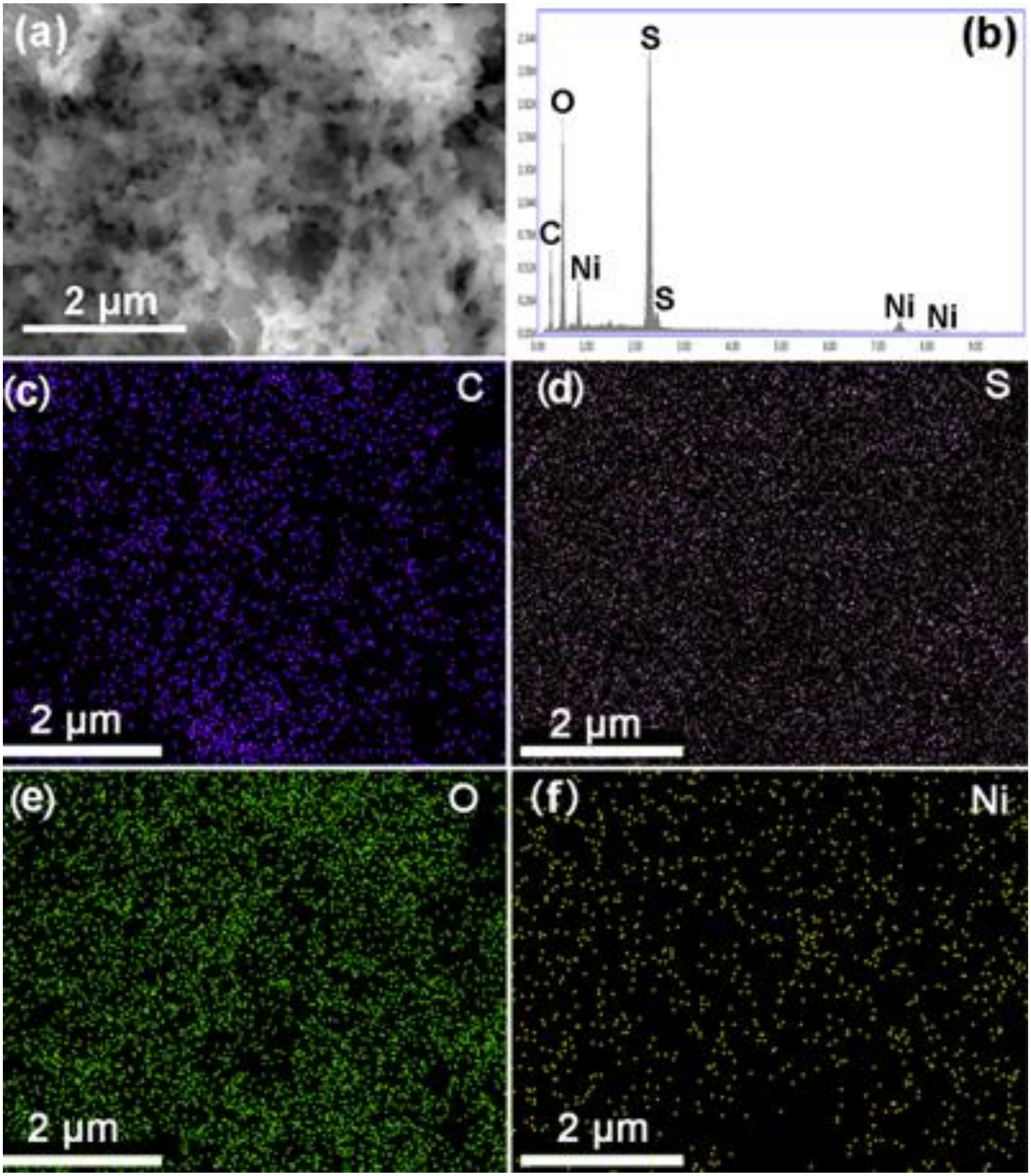

Figure S22 SEM image (a), EDX spectrum (b) of S/CNF-HC-Ni_3S_2 electrode and corresponding elemental maps with C (c), S (d), O (e), and Ni (f) after 100 cycles.

References

[S1] C. Ye, L. Zhang, C. Guo, D. Li, A. Vasileff, H. Wang, S. Qiao, *Adv. Funct. Mater.* **2017**, *27*, 1702524.

[S2] C. Li, J. Shi, L. Zhu, Y. Zhao, J. Lu, L. Xu, *Nano Res.* **2018**, *11*, 4302.

[S3] D. Lv, J. Zheng, Q. Li, X. Xie, S. Ferrara, Z. Nie, L. Mehdi, N. Browning, J. Zhang, G. Graff, *Adv. Energy Mater.* **2015**, *5*, 1402290.

[S4] Q. Zhao, Q. Zhu, J. Miao, Z. Guan, H. Liu, R. Chen, Y. An, F. Wu, B. Xu, *ACS Appl. Mater. Interfaces* **2018**, *10*, 10882.

[S5] Q. Pang, D. Kundu, L. Nazar, *Mater. Horiz.* **2016**, *3*, 130.

[S6] Z. Ma, L. Tao, D. Liu, Z. Li, Y. Zhang, Z. Liu, H. Liu, R. Chen, J. Huo, S. Wang, *J. Mater. Chem. A* **2017**, *5*, 9412.

[S7] Z. Li, S. Zhang, J. Zhang, M. Xu, R. Tatara, K. Dokko, M. Watanabe, *ACS Appl. Mater. Interfaces* **2017**, *9*, 38477.

[S8] L. Zhang, Z. Chen, N. Dongfang, M. Li, C. Diao, Q. Wu, X. Chi, P. Jiang, Z. Zhao, L. Dong, *Adv. Energy Mater.* **2018**, *8*, 1802431.

[S9] Q. Pang, C. Y. Kwok, D. Kundu, X. Liang, L. Nazar, *Joule* **2019**, *3*, 136.

[S10] W. Chen, T. Lei, T. Qian, W. Lv, W. He, C. Wu, X. Liu, J. Liu, B. Chen, C. Yan, *Adv. Energy Mater.* **2018**, *8*, 1702889.

[S11] H. Yuan, H. Peng, B. Li, J. Xie, L. Kong, M. Zhao, X. Chen, J. Huang, Q. Zhang, *Adv. Energy Mater.* **2019**, *9*, 1802768.

[S12] G. Xu, Q. Yan, P. Bai, H. Dou, P. Nie, X. Zhang, *ChemistrySelect* **2019**, *4*, 698.

[S13] Y. Zhong, X. Xia, S. Deng, J. Zhan, R. Fang, Y. Xia, X. Wang, Q. Zhang, J. Tu, *Adv. Energy Mater.* **2018**, *8*, 1701110.

[S14] L. Kong, X. Chen, B. Li, H. Peng, J. Huang, J. Xie, Q. Zhang, *Adv. Mater.* **2018**, *30*, 1705219.

[S15] J. Zhang, Z. Li, Y. Chen, S. Gao, X. W. Lou, *Angew. Chem. Int. Ed.* **2018**, *57*, 10944.

[S16] Y. Zhong, D. Chao, S. Deng, J. Zhan, R. Fang, Y. Xia, Y. Wang, X. Wang, X. Xia, J. Tu, *Adv. Funct. Mater.* **2018**, *28*, 1706391.

[S17] W. Cai, G. Li, K. Zhang, G. Xiao, C. Wang, K. Ye, Z. Chen, Y. Zhu, Y. Qian, *Adv. Funct. Mater.* **2018**, *28*, 1704865.

[S18] L. Kong, B. Li, H. Peng, R. Zhang, J. Xie, J. Huang, Q. Zhang, *Adv. Energy Mater.* **2018**, *8*, 1800849.

[S19] Y. Wang, R. Zhang, Y. Pang, X. Chen, J. Lang, J. Xu, C. Xiao, H. Li, K. Xi, S. Ding, *Energy Storage Mater.* **2019**, *16*, 228.

[S20] Y. You, Y. Ye, M. Wei, W. Sun, Q. Tang, J. Zhang, X. Chen, H. Li, J. Xu, *Chem. Eng. J.* **2019**, *355*, 671.
